# Machine Learning Approaches for the Image-Based Identification of Surgical Wound Infections: Scoping Review

**DOI:** 10.2196/52880

**Published:** 2024-01-18

**Authors:** Juan Pablo Tabja Bortesi, Jonathan Ranisau, Shuang Di, Michael McGillion, Laura Rosella, Alistair Johnson, PJ Devereaux, Jeremy Petch

**Affiliations:** 1 Centre for Data Science and Digital Health Hamilton Health Sciences Hamilton, ON Canada; 2 Dalla Lana School of Public Health University of Toronto Toronto, ON Canada; 3 Population Health Research Institute Hamilton, ON Canada; 4 SickKids Research Institute Toronto, ON Canada; 5 Institute for Health Policy, Management and Evaluation University of Toronto Toronto, ON Canada; 6 Division of Cardiology McMaster University Hamilton, ON Canada

**Keywords:** surgical site infection, machine learning, postoperative surveillance, wound imaging, mobile phone

## Abstract

**Background:**

Surgical site infections (SSIs) occur frequently and impact patients and health care systems. Remote surveillance of surgical wounds is currently limited by the need for manual assessment by clinicians. Machine learning (ML)–based methods have recently been used to address various aspects of the postoperative wound healing process and may be used to improve the scalability and cost-effectiveness of remote surgical wound assessment.

**Objective:**

The objective of this review was to provide an overview of the ML methods that have been used to identify surgical wound infections from images.

**Methods:**

We conducted a scoping review of ML approaches for visual detection of SSIs following the JBI (Joanna Briggs Institute) methodology. Reports of participants in any postoperative context focusing on identification of surgical wound infections were included. Studies that did not address SSI identification, surgical wounds, or did not use image or video data were excluded. We searched MEDLINE, Embase, CINAHL, CENTRAL, Web of Science Core Collection, IEEE Xplore, Compendex, and arXiv for relevant studies in November 2022. The records retrieved were double screened for eligibility. A data extraction tool was used to chart the relevant data, which was described narratively and presented using tables. Employment of TRIPOD (Transparent Reporting of a Multivariable Prediction Model for Individual Prognosis or Diagnosis) guidelines was evaluated and PROBAST (Prediction Model Risk of Bias Assessment Tool) was used to assess risk of bias (RoB).

**Results:**

In total, 10 of the 715 unique records screened met the eligibility criteria. In these studies, the clinical contexts and surgical procedures were diverse. All papers developed diagnostic models, though none performed external validation. Both traditional ML and deep learning methods were used to identify SSIs from mostly color images, and the volume of images used ranged from under 50 to thousands. Further, 10 TRIPOD items were reported in at least 4 studies, though 15 items were reported in fewer than 4 studies. PROBAST assessment led to 9 studies being identified as having an overall high RoB, with 1 study having overall unclear RoB.

**Conclusions:**

Research on the image-based identification of surgical wound infections using ML remains novel, and there is a need for standardized reporting. Limitations related to variability in image capture, model building, and data sources should be addressed in the future.

## Introduction

Postoperative complications are associated with significant morbidity and mortality [1,2]. Wound-related issues following surgery remain common and represent a considerable cost to patients and health care systems [3,4]. The global incidence of surgical site infections (SSIs)—which include superficial or deep infections occurring at the incision site as well as organ-space infections related to the surgery [5]—has been estimated to be 11% [6]. Many of these events occur after hospital discharge, highlighting the need for remote posthospital discharge monitoring. Early research suggests that remote postoperative wound follow-up is associated with high patient satisfaction and reduced costs [7,8].

Artificial intelligence tools have been applied to various aspects of health care and are contributing to the shift toward precision medicine [9-11]. Specifically, machine learning (ML) techniques can leverage health data and develop predictive models to assist in clinical decision-making [12], and can be used in conjunction with computer vision. An important medical task is the classification and detection of various objects, ranging from skin lesions to cell nuclei [13]. Recently, ML-enabled computer vision methods have been used to contribute to the automation of wound segmentation [14,15], evaluation of postoperative outcomes [16,17], and improvement of wound assessment practices [18,19], often outperforming existing approaches.

Wound care involves cleaning and dressing, monitoring healing, addressing possible infection, and other wound type-specific measures [20]. Current image-based wound management practices, often involving manual wound photography and assessment carried out by nurses, are time- and labor-intensive [21]. In contrast, models of care augmented with ML-enabled methods can be automated [22,23]. The portability of these methods might also be employed to conduct such assessments remotely [24], reducing patient travel burden and improving access to wound care in rural areas [25,26]. A recent clinical trial (Post-Discharge After Surgery Virtual Care With Remote Automated Monitoring-1) found that virtual care with remote monitoring that included wound evaluation shows promise in improving outcomes important to patients and to optimal health system function [27]. These results highlight the utility of digital approaches to care, which can be integrated with automated ML systems to increase scalability.

The research landscape of ML-based methods for wound surveillance is evolving rapidly. Several reviews have addressed the use of ML for various aspects of wound care from different perspectives. One scoping review focused on mapping the use cases for ML in the management of various types of chronic wounds (eg, visual assessment and predicting evolution) [28]. Another review addressed image-based chronic wound assessment from a technical standpoint, characterizing existing rule-based and ML methods for wound feature extraction and classification, as well as systems for wound imaging [29]. However, chronic and acute wounds differ in terms of the clinical signs associated with infection as those in chronic wound infections are often less discernible [30], and there is a need to establish the state of the science with respect to how ML-based tools are being used for postoperative wounds. One systematic review specifically characterized the effectiveness of ML algorithms that use textual or structured data for the detection and prediction of SSIs [31], though a survey of image-based methods has not been undertaken. Likewise, other systematic reviews have found that reporting in ML-based prediction model studies is generally poor and that most are at high risk of bias (RoB) [32,33]. Considering these results, assessments of RoB and the employment of reporting guidelines—which have not been included in previous reviews of image-based ML for wound care—can further provide insights into the current state of research in this field.

The scope and purpose of this review was to provide an in-depth overview of ML approaches that use visual data for the identification of SSIs. Specifically, this review describes the nature of the methods used in this context, the ways in which they have been validated, the extent to which the reporting of these studies follows guideline recommendations, and their RoB.

## Methods

### Review Methodology

This scoping review was conducted in accordance with the appropriate JBI (Joanna Briggs Institute) methodology [34]. The PRISMA-ScR (Preferred Reporting Items for Systematic Reviews and Meta-Analyses extension for Scoping Reviews) checklist was used to guide the writing of this review [35]. We opted for a scoping review approach as we sought to analyze the methods employed in conducting research in this field, an indication for scoping reviews [36], rather than synthesize model performance.

### Search Strategy and Study Selection

Following our protocol [37], participants of any age (or other demographic variable) who underwent any type of surgery were considered. The main concept being addressed is the use of ML-based computer vision in the image-based identification of surgical wound infections. Only wounds that were directly the product of surgery were included. Other types of wounds, such as pressure ulcers, were excluded. We included studies that described detection of infection of such wounds (as defined by study authors). Studies solely focusing on tasks other than identification (eg, segmentation) and using sources other than images or videos for prediction were not considered. Studies conducted in any postoperative context, including postdischarge settings, were included.

Studies that developed or validated one or more prediction models were included in this review, including those that gathered data from experimental, quasi-experimental, and observational studies (eg, randomized controlled trials, and prospective and retrospective studies). Only primary sources were considered. Select grey literature sources, such as conference proceedings and preprints, were also considered. Animal studies were excluded.

An initial limited search of MEDLINE (Ovid) and CINAHL (EBSCO) was undertaken to identify relevant papers. Text words used in the titles and abstracts of retrieved records, as well as index terms used to describe them, were used to develop the full search strategy (Multimedia Appendix 1), which was adapted for each database. The databases we searched were MEDLINE (Ovid), CENTRAL (Ovid), Embase (Ovid), CINAHL (EBSCO), Web of Science Core Collection, IEEE Xplore, and Compendex. We also searched arXiv for relevant preprints. All databases were searched from inception to November 24, 2022. Reference lists of all included records were likewise searched for other records. Only English-language records were considered.

After the search was completed, duplicate citations were removed and all identified citations were uploaded into Rayyan [38] for title and abstract and full-text screening by 2 independent reviewers. An abstract screening tool was used to aid in the screening process (Multimedia Appendix 2). The texts of potentially relevant records were retrieved in full and assessed in the same manner. Disagreements were resolved through discussion or by consultation with an additional reviewer.

### Assessment of the Employment of Reporting Guidelines and RoB

A data extraction tool (Multimedia Appendix 3)—that had been piloted with 20% (2/10) of the included reports by 2 independent reviewers—was used to abstract the relevant data. After piloting the tool, a single reviewer extracted data from the remaining sources with validation by an additional reviewer. The data were summarized using tables and presented narratively.

We determined the extent to which the included reports employed TRIPOD (Transparent Reporting of a Multivariable Prediction Model for Individual Prognosis or Diagnosis) guidelines using the TRIPOD adherence assessment form [39], and used the PROBAST (Prediction Model Risk of Bias Assessment Tool) to conduct critical appraisal [40]. Further, 2 reviewers assessed both employment of reporting guidelines and RoB for 20% (2/10) of the included reports; the remaining assessments were carried out by 1 reviewer (with an additional reviewer available for validation). In studies that developed multiple models, we only evaluated reporting and RoB for those that were image-based. To facilitate comparison between the reporting level of TRIPOD items, we chose arbitrary thresholds to denote high (≥70%), moderate (40%-69%), and low (1%-39%) adherence.

The TRIPOD adherence form and PROBAST were modified as needed for the purposes of this review. As has been noted in other reviews [33,41-43], it is difficult to assess RoB in the predictors of deep learning (DL) models that use images for prediction, as the image features are automatically selected by the algorithm. Still, we deemed image capture considerations important (eg, whether images were systematically captured) and altered the relevant TRIPOD and PROBAST items accordingly. The full list of modifications can be found in Multimedia Appendix 4.

## Results

### Study Inclusion

**Figure 1:**
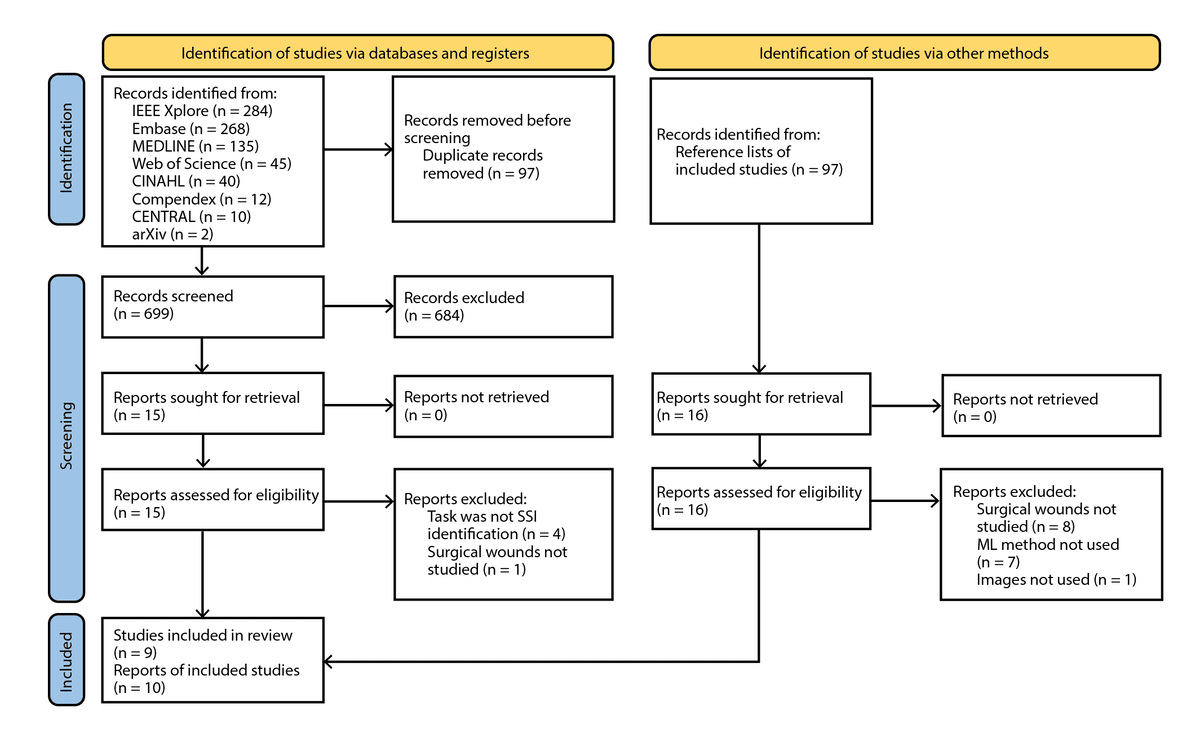
PRISMA flow diagram showing study selection process. ML: machine learning; PRISMA: Preferred Reporting Items for Systematic Reviews and Meta-Analyses; SSI: surgical site infection.

The search retrieved 796 records, or 699 records after duplicates were removed (Figure 1). We excluded 684 records during initial screening and full-text screened 15 reports. We identified 10 reports that met the eligibility criteria. The reference lists of these reports had an additional 16 potentially relevant records, though none met the eligibility criteria.

### Review Findings

The included studies took place in a variety of settings, across a wide range of cohort sizes (Table 1). Important study characteristics were sometimes unclear or not reported. The full data extraction sheet can be found in Multimedia Appendix 5.

**Table 1:** Study characteristics.

Author	Purpose	Setting	Cohort	Events
Fletcher et al [44]	To develop a model for predicting SSI^a^ in C-section wounds from thermal images taken with smartphones	Women who underwent C-section at a particular hospital in Kigali, Rwanda, between September 2019 and February 2020 prospectively enrolled on postoperative day 1	In total, 530 participants	In total, 30 participants with infected wounds
Fletcher et al [45]	To develop a model for predicting SSI in C-section wounds from color images taken with mobile devices	Women aged >18 years who underwent C-section at a particular hospital in Kigali, Rwanda, between March and October 2017 enrolled prior to discharge	In total, 572 participants (out of 729) that returned for follow-up	In total, 62 participants with infected wounds
Wu et al [46]	To develop an automatic monitoring tool for surgical wounds based on smartphone images	Prospectively collected wound database of patients who had undergone laparotomy, minimal invasive surgery, or hernia repair at an Asian academic center	In total, 480 wound images from 100 patients	In total, 136 images of infected wounds
Fletcher et al [47]	To develop models for predicting SSI in C-section wounds from questionnaire and image data	Women aged 18+ years who underwent C-section at a particular hospital in Kigali, Rwanda, between March and October 2017 enrolled prior to discharge	In total, 572 participants (out of 729) that returned for follow-up; images available for 568 patients	In total, 62 participants with infected wounds
Hsu et al [48]	To develop an automatic wound interpretation app for automated wound monitoring	Images of chest, abdomen, back, hand, and podiatry wounds collected from the Department of Surgery and Department of Internal Medicine of National Taiwan University Hospital	In total, 293 wound images	In training set, 27 infection images; total number unclear
Lüneburg et al [49]	To explore ML^b^ approaches for remote LVAD^c^ patient monitoring using images	Images of LVAD driveline exit sites obtained from Schüchtermann-Schiller’sche Kliniken and Hannover Medical School	In total, 745 images from 61 patients, though only 732 are labeled	In total, 212 images of mild infection and 37 images of severe infection
Shenoy et al [50]	To develop a model that can identify the onset of wound ailments from smartphone images	Images collected primarily from patients and surgeons at the Palo Alto Veterans Affairs Hospital and the Washington University Medical Center	In total, 1335 images	In total, 355 images of infection
Hsu et al [51]	To develop a model for recognizing SSI	Images collected from the Department of Surgery of National Taiwan University Hospital	In total, 42 images	In total, 30 images of infection
Zeng et al [52]	To develop a system for automatic wound detection and subsequent infection detection	Not reported	Total unclear; 6 images for testing	Unclear
Wang et al [53]	To develop an integrated system for automatic wound segmentation and analysis of wound conditions from wound images	Images collected from New York University Wound Database	In total, 3400 images	In total, 155 images of infection

^a^SSI: surgical site infection.

^b^ML: machine learning.

^c^LVAD: left ventricular assist device.

The earliest included paper was published in 2015 [53], 6 papers were published between 2017 and 2019 [47-52], and 3 papers were published between 2020 and November 2022 [44-46].

The objective of the included studies was generally to develop models for identifying surgical wound infection from images. In some cases, the purpose was broader; 2 studies sought to identify the presence of various wound attributes (eg, granulation) [46,50] and 4 studies developed models for automatic wound segmentation [48,49,51,53]. Other objectives included healing progress prediction [53], surface area estimation [53], and wound detection [52].

### Patients, Procedures, and Image Capture

The types of patients and surgical procedures studied varied. In total, 3 papers focused on C-section patients in rural Rwanda [44,45,47], while another study examined patients implanted with a left ventricular assist device in Germany [49]. Further, 2 studies conducted in Asia described the surgical procedures more broadly; for instance, 1 paper included patients that had undergone laparotomy, minimal invasive surgery, or hernia repair [46], while another included surgical wounds of the chest, abdomen, back, hands, and feet [48]. In 4 papers, this information was not specified [50-53].

The context of image capture likewise varied (Table 2). Most studies simply stated that images were obtained from one or more sites or data sets [48-51,53], without further details on how the images were selected; though 1 study additionally indicated that the data were “prospectively collected” [46] and the studies conducted in Rwanda described their cohorts in the greatest detail [44,45,47].

**Table 2:** Study data collection and ML^a^ methodology.

Author	Time of image capture	Imaging modality	Outcome determination	Modeling methods	Performance metric for best-performing model
Fletcher et al [44]	Approximately 10 days after surgery	Thermal images taken by community health workers with a thermal camera module connected to smartphone that produces a JPG thermal image and a separate 2D temperature array	Physical examination performed by general practitioner	CNN^b^	Median AUC^c^: 0.90
Fletcher et al [45]	Approximately 10 days after surgery	Color images taken by community health workers with Android tablets	Physical examination performed by general practitioner	CNN	Median AUC: 0.655
Wu et al [46]	Just after surgery, during hospitalization, and in outpatient clinic follow-up	Color images taken by surgeons with smartphones	Annotation of abnormal wound features on images performed by surgeons	CNN, SVM^d^, RF^e^, GB^f^	Median AUC: 0.833
Fletcher et al [47]	Approximately 10 days after surgery	Color images taken by community health workers with Android tablets	Physical examination performed by general practitioner	Unclear; potentially both SVM and logistic regression	Median AUC: 1.0
Hsu et al [48]	Not reported	Color images taken with smartphones	Unclear, but likely annotation of images by 3 physicians	SVM	Overall accuracy: 0.8358
Lüneburg et al [49]	Not reported	Color images; device not reported	Unclear, but likely based on physical examination performed by physicians	CNN	Overall accuracy: 0.670
Shenoy et al [50]	Not reported	Color images taken by patients and surgeons with smartphones	Not reported	CNN	AUC: 0.82
Hsu et al [51]	Not reported	Color images; device not reported	Not reported	SVM	Overall accuracy: 0.9523
Zeng et al [52]	Not reported	Color images; device not reported	Not reported	SVM	AUCs varied depending on the infection-related wound attribute, but ranged from 0.7682 to 0.9145.
Wang et al [53]	Not reported	Color images; device not reported	Not reported	SVM using CNN features	AUC: 0.847

^a^ML: machine learning.

^b^CNN: convolutional neural network.

^c^AUC: area under curve.

^d^SVM: support vector machine.

^e^RF: random forest.

^f^GB: gradient boosting.

In the studies conducted with C-section patients, the wounds were photographed approximately 10 days after surgery, with infection assessment taking place on the same day [44,45,47]. Another study collected images at multiple time points: immediately after surgery, during hospitalization, and at a later follow-up, though the number of days post surgery was not indicated [46]. However, the time at which the images were taken relative to surgery and the time at which infection was assessed relative to image capture were not reported in 6 records [48-53].

In terms of the images themselves, 9 studies used color images [45-53], and 1 used thermal images [44]. Further, 6 studies used a mobile device (either smartphone or tablet) to capture the image [44-48,50], while others did not report the device used [49,51-53]. Across studies that reported the persons responsible for capturing the images, community health workers were typically responsible [44,45,47]; 1 study used images taken by surgeons [46]; and another used images collected by both patients and surgeons [50].

Assessment of surgical wound infection establishes the model ground truth and occurred mainly through face-to-face physical examination [44,45,47,49], through manual annotation of the wound images [46,48,51], or was not reported [50,52,53].

### ML Approaches

All the included records were model development studies (ie, no external validation). In total, 4 papers used convolutional neural networks (CNNs) [44,45,49,50], 3 used support vector machines (SVMs) developed using handcrafted features [48,51,52], 1 trained an SVM classifier using CNN-derived features [53], 1 used a CNN, an SVM, a random forest model, and a gradient boosting classifier [46], and 1 paper’s methods were not entirely clear but may have involved both logistic regression and SVMs [47]. Additional technical details are available in Multimedia Appendix 6.

The number of images used for developing an infection detection model ranged from just 42 [51] to 3400 [53]. Likewise, the proportion of images of infected wounds ranged from 4.6% (155/3400) [53] to 71.4% (30/42) [51]. In some cases, there was 1 image per patient [44,45,47], while in others, there were multiple per patient [46,49] or the number of patients was not reported [48,50-53].

In 5 papers, the classification task was binary [44-47,53], while in most others, the task was multiclass. In 1 paper, multiclass classification entailed distinguishing between mild, severe, and no infection [49], while in 3 others, the model differentiated between various infection-related wound attributes, such as granulation and swelling [48,51,52]. In contrast, 1 paper addressed a multilabel task in which the model identified the presence of a wound, infection, granulation, and drainage per image [50].

All studies reported model performance. In total, 7 studies reported area under the receiver operating characteristic curve values, which ranged from 0.655 [45] to 1.0 [47] for the best-performing models. The remaining studies reported overall accuracies, ranging from 0.670 [49] to 0.952 [51] for the best-performing models, as well as other performance metrics (eg, *F*_1_-scores).

### Employment of Reporting Guidelines

There were a few TRIPOD items that were highly employed (ie, employed by at least 7 out of the 10 included studies). For instance, all papers reported their objectives, and most reported background information, overall interpretations of study results, and descriptions of whether actions were taken to standardize image capture or otherwise systematically identify wounds from the images. In addition, 6 TRIPOD items had moderate employment (employed by between 4 and 6 studies); namely, the reporting of data sources and study setting, descriptions of model-building procedures, the number of participants or images (and the number showing infection), study limitations, as well as the potential clinical use of the models and future research directions.

Employment of 8 TRIPOD items was low (employed by between 1 and 3 studies), including items related to the reporting of participant selection methods, descriptions of how and when images were taken, rationales for sample sizes, the flow of participants within the paper, explanations of how to use the models, and funding details. Most studies did not completely use these guidelines in terms of outcome assessment, as there was often no indication of the criteria used to diagnose surgical wound infection or the time interval between surgery and assessment was unclear.

An additional 7 TRIPOD items were not reported in any of the included studies. Titles and abstracts did not employ reporting guidelines, and participant demographics were not reported. Similarly, model calibration was not discussed, and in studies that did not exclusively use DL methods for infection detection [46-48,51-53], reporting of feature modeling details did not meet TRIPOD guidelines.

### About RoB

The RoB assessment led to 9 studies being identified as having an overall high RoB, while the remaining study was determined to have overall unclear RoB (Table 3). The participants domain was determined to be unclear in terms of RoB because little information about the source of data and recruitment methods was reported [46,48-53]. The 3 papers on C-section patients in Rwanda were at low RoB for this domain, as the nature of these works was cross-sectional and the cohorts were well defined [44,45,47]. In terms of predictors, we identified 5 papers as being at high RoB since there was variability in image capture conditions without later accounting for this variability [45-47,50,53]. In contrast, other papers were judged to be at low RoB for this domain because they segmented the wound prior to infection detection [48,49,51] or placed a frame around the wound prior to image capture [44], improving the uniformity of images processed for model training. Likewise, most studies were rated as having unclear RoB in the outcome domain, largely because the specific criteria used to gauge the presence of surgical wound infection were not reported. In other cases, the outcome domain was determined to be at high RoB because the presence of infection was determined solely from images, as opposed to by face-to-face review. In 8 studies, the analysis domain was assessed as being at high RoB for many reasons [45,47-53], including omission of participants in model development, an absence of both discrimination and calibration measures, and failure to appropriately account for overfitting.

**Table 3:** PROBAST^a^ RoB^b^ assessment of the 10 included reports.

Study	RoB				
	Participants	Predictors	Outcome	Analysis	Overall
Fletcher et al [44]	+^c^	+	?^d^	?	?
Fletcher et al [45]	+	−^e^	?	−	−
Wu et al [46]	?	−	−	?	−
Fletcher et al [47]	+	−	?	−	−
Hsu et al [48]	?	+	−	−	−
Lüneburg et al [49]	?	+	?	−	−
Shenoy et al [50]	?	−	?	−	−
Hsu et al [51]	?	+	−	−	−
Zeng et al [52]	?	?	?	−	−
Wang et al [53]	?	−	?	−	−

^a^PROBAST: Prediction Model Risk of Bias Assessment Tool.

^b^RoB: risk of bias.

^c^+ indicates low RoB.

^d^? indicates unclear RoB.

^e^− indicates high RoB.

## Discussion

### Principal Findings

This scoping review aimed to characterize the available research on ML approaches for the image-based identification of surgical wound infections. Such research is important as it can be integrated with remote patient monitoring, which enables improved health care decision-making and management, with additional benefits such as reduced travel burden. Initial work has suggested that remote image-based monitoring of wounds is feasible and associated with higher patient satisfaction [54-56], and is at least comparable to routine in-person care in terms of time to infection diagnosis [57]. Other aspects of wound assessment targeted by image-based remote patient monitoring include identification of dehiscence and surface area and temperature measurements [58,59], though much has not been automated or ML-based.

Despite the extensive body of ML-based work using medical images in other specialties [60,61], there is scarce ML research on the identification of surgical wound infections from digital images. We identified only 10 such papers, 7 of which were conference papers, which limits the space for reporting and likely contributed to the low reporting of TRIPOD items. In contrast, a recent review of ML for SSI detection identified 32 papers that used structured electronic health record, free-text, or administrative data for prediction [31], suggesting that ML-based SSI detection research has mostly used these more readily available data sources. While models based on such in-hospital data perform well in the context of inpatient SSI detection, they may be limited in their practical application during clinical care, as visual inspection is the essential mode by which infection is identified. In terms of incorporating innovative imaging techniques, thermal imaging has recently emerged as a potentially valuable tool in the management of surgical wounds [62-64]. Thermal imaging can be used with mobile devices [44,65], which facilitates its application for postdischarge monitoring, and may better generalize to different skin colors. On the other hand, the utility of electronic health record– or text-based models for postdischarge surveillance is perhaps less clear. Current postdischarge surgical wound surveillance largely depends on evaluation at follow-up visits, which may be infrequent and not timely [66], or on patient self-assessment, which is not reliable [67,68]. ML for the image-based identification of surgical wound infections presents the opportunity to automate this practice.

### Reporting Data Collection Details

ML hinges on effective data collection, which can be challenging in outpatient or remote monitoring settings; hence, this type of research is still in early development. Although virtual care as a model of health care is relatively new, progress has been made in terms of data collection technology [64,69], similar telemedicine research without ML [70-72], and monitoring of other wound types [73,74]. As almost three-fourths of individuals worldwide own a mobile phone [75], leveraging this technology for remote monitoring holds potential. Still, it is worth noting that mobile phone ownership and mobile network coverage is lower in certain geographical areas and in low-income groups. In these contexts, alternative approaches, such as in-hospital follow-up with pictures taken by a community health worker [44], may be more appropriate. In terms of the data used in the included studies, it has mainly been collected in non-Western settings, and there are no publicly available data sets of surgical wound infection images, which presents a challenge to reproducibility and further development in the field. Likewise, the lack of reporting on image metadata (eg, gender and age distributions, procedures received, and occurrence of surgical complications) and eligibility criteria limits the understanding of the populations that this research can be generalized to and contributes to RoB in terms of participant selection. Reporting of such details needs improvement for the progression of different prototypes for different subpopulations in this domain.

### Transparency and Standardization in Model Development

The nature of the models developed in the included studies was diagnostic rather than prognostic. Similarly, none of the included papers performed out-of-sample external validation, highlighting the newness of this field of work and opportunity for further maturity. Interestingly, 4 papers published between 2017 and 2019 did not use DL methods, perhaps because the expertise required for development of such models was not yet widely available. Model performance is likewise not well-standardized in its reporting, as no papers reported on calibration and some did not include discrimination measures, which gives rise to RoB in analysis methods. Many papers did not report on measures to address overfitting, which calls the developed models’ generalizability into question. Despite the partial reporting, the performance of the models in the included papers suggest that image-based ML for identification of surgical wound infection holds promise. In order to better understand their generalizability and reliability, future studies should externally validate and calibrate the developed models and report areas under the curve (as opposed to solely reporting other measures such as accuracy), and provide transparent documentation (eg, open-source code) to promote reproducibility and collaboration. Considering that interpretability and explainability support clinician trust [76], researchers may likewise wish to explore these concepts in future work.

### Employment of Reporting Guidelines and RoB

#### Standardization of Image Capture

Use of TRIPOD guidelines was mostly low and RoB was generally unclear or high. This was in part due to participant- and analysis-related considerations discussed above; however, there were also concerns with the images themselves. In most studies, the way in which the images were taken, environmental conditions, the persons responsible for taking them, and the time of image capture relative to surgery, were not reported in detail. Still, there was often variability in the conditions of image capture, which might be attributed to unique challenges associated with collection and standardization in this particular modality. As opposed to other modalities, surgical wound infection images are largely taken by hand, without explicit training or guidance, which makes for considerable differences among images and introduces RoB in terms of model predictors. Efforts to standardize image capture help reduce RoB by minimizing systematic differences between images of infected versus noninfected wounds. Recent approaches such as instructions for patient-generated surgical wound images [77] or automated color calibration, scaling, and rotation correction [78] suggest that these considerations are receiving attention. Some studies created segmentation algorithms to capture the wound more reliably from the nonuniform images, which may have hindered the development of infection detection models. Segmentation and classification represent distinct areas of research, though many studies developed their own segmentation models rather than using or building on existing segmentation algorithms. In future work, specific directions detailing the time (relative to surgery), method, and conditions of image capture should be provided in order to reduce unwanted variability, and image processing steps can be undertaken for further standardization.

#### Transparency in Outcome Assessment

Outcome assessment was also not well reported in most papers. While there is no universally accepted and objective gold standard for SSI detection [79], clinical examination (involving direct observation of the wound) is frequently used as a reference standard [68,70,72,80,81]. Although some studies did perform in-person clinical examination, none reported the specific criteria used to gauge the presence of infection. Considering that there are differences in the rates of reported SSIs depending on the criteria used [82], specifying these criteria is important to more accurately assess the RoB arising from outcome assessment. It is worth noting, however, that there are challenges associated with in-person postoperative wound assessment. Surgical wound infections progress variably, with some only apparent after the 30-day postoperative surveillance benchmark [83,84]. However, extended in-person follow-up timeframes may require additional administrative resources. In practice, the criteria employed for SSI assessment typically consider both feasibility and validity [79]. This may necessitate striking a balance between resources, time constraints, and quality of assessment, which can pose challenges to the comprehensive evaluation of surgical wound infections. On a smaller scale, interrater reliability of in-person SSI assessment using established criteria can be modest [85,86], and in rural areas, there may be limited access to high-quality in-person wound care. Where feasible, determination of ground truth should use established criteria for infection and employ multiple independent assessors to minimize RoB.

### Limitations

There are some limitations to this review. For instance, additional searching (eg, forward citation searching) may have led to more relevant reports being identified, as may have searching grey literature sources, which would reduce selection bias. We may have missed other relevant non–English-language papers, potentially excluding valuable studies. The included studies are from diverse locations (eg, Rwanda, Germany, and Taiwan), though this does not fully compensate for the potential language bias. Similarly, data extraction and the TRIPOD and PROBAST assessments were mainly completed by 1 reviewer, which introduces a potential source of bias in our findings. The modifications made to the TRIPOD and PROBAST tools may limit the ability to compare the results of our assessments to those of other reviews. Artificial intelligence–oriented extensions of both tools are in development [87] and will facilitate their use in appraising ML-based studies.

### Conclusions

The use of ML for the image-based identification of surgical wound infections remains in the early stages, with only 10 studies available and a need for reporting standardization. Future development and validation of such models should carefully consider image variability, overfitting concerns, and criteria for determination of infection. These considerations are important to advance the state of image-based ML for wound management, which has the potential to automate traditionally labor-intensive practices.

## Appendix

Multimedia Appendix 1 Search strategy.

Multimedia Appendix 2 Abstract screening tool.

Multimedia Appendix 3 Data extraction tool.

Multimedia Appendix 4 TRIPOD and PROBAST altered or excluded items.

Multimedia Appendix 5 Complete results of data extraction, assessment of TRIPOD employment, and PROBAST assessment.

Multimedia Appendix 6 Additional technical details.

Multimedia Appendix 7 PRISMA-ScR checklist.

## Abbreviations

CNN: convolutional neural network
DL: deep learning
JBI: Joanna Briggs Institute
ML: machine learning
PRISMA-ScR: Preferred Reporting Items for Systematic Reviews and Meta-Analyses Extension for Scoping Reviews
PROBAST: Prediction Model Risk of Bias Assessment Tool
RoB: risk of bias
SSI: surgical site infection
SVM: support vector machine
TRIPOD: Transparent Reporting of a Multivariable Prediction Model for Individual Prognosis or Diagnosis
