# Supplementary material for: Machine Learning Approaches for the Image-Based Identification of Surgical Wound Infections: Scoping Review
Source: J Med Internet Res. 2024 Jan 18;26:e52880. doi: 10.2196/52880 (PMC10835585; doi:10.2196/52880)
Supplement: Multimedia Appendix 1 [file jmir_v26i1e52880_app1.docx]

# Multimedia Appendix 1. Search strategy.

**MEDLINE**

| Ovid MEDLINE(R) ALL <1946 to Present> (date searched: November 24, 2022): | | |
| --- | --- | --- |
|  |  |  |
| 1 | (machine learning or artificial intelligence or AI).mp. or Artificial Intelligence/ or exp Machine Learning/ or exp Expert Systems/ or exp Pattern Recognition, Automated/ | 183121 |
| 2 | exp Neural Networks, Computer/ or (neural network* or deep learning).mp. | 116391 |
| 3 | (support vector machine* or SVM or decision tree* or gradient boost* or random forest*).mp. | 61793 |
| 4 | (computer vision or machine vision).mp. | 8021 |
| 5 | 1 or 2 or 3 or 4 | 291816 |
| 6 | exp Perioperative Medicine/ or exp Perioperative Care/ or exp Perioperative Period/ or (periop* or peri-op*).mp. | 349989 |
| 7 | exp Postoperative Complications/ or (postop* or post-op*).mp. | 1147633 |
| 8 | (post-surg* or post-discharge).mp. | 29953 |
| 9 | 6 or 7 or 8 | 1295146 |
| 10 | wound*.mp. or exp "Wounds and Injuries"/ | 1242717 |
| 11 | exp Wound Healing/ | 140530 |
| 12 | exp Wound Infection/ or (surgical site infection* or SSI*).mp. | 63294 |
| 13 | 10 or 11 or 12 | 1270484 |
| 14 | exp Image Processing, Computer-Assisted/ or exp Image Interpretation, Computer-Assisted/ or imag*.mp. | 2823565 |
| 15 | exp Photography/ or exp Video Recording/ or (photo* or video*).mp. | 1614151 |
| 16 | 14 or 15 | 3678192 |
| 17 | 5 and 9 and 13 and 16 | 135 |
| 18 | remove duplicates from 17 | 135 |

**Embase**

| Embase Classic+Embase <1947 to 2022 November 23> | | |
| --- | --- | --- |
|  |  |  |
| 1 | (machine learning or artificial intelligence or AI).mp. or exp artificial intelligence/ or exp machine learning/ or exp expert system/ or exp automated pattern recognition/ | 433572 |
| 2 | exp artificial neural network/ or (neural network* or deep learning).mp. | 146915 |
| 3 | (support vector machine* or SVM or random forest* or gradient boost* or decision tree*).mp. | 83315 |
| 4 | exp computer vision/ or (computer vision or machine vision).mp. | 9005 |
| 5 | 1 or 2 or 3 or 4 | 498030 |
| 6 | exp perioperative medicine/ or exp perioperative care/ or exp perioperative period/ or (periop* or peri-op*).mp. | 213686 |
| 7 | exp postoperative period/ or exp postoperative complication/ or exp postoperative care/ or (postop* or post-op*).mp. | 1852773 |
| 8 | (post-surg* or post-discharge).mp. | 58917 |
| 9 | 6 or 7 or 8 | 1977349 |
| 10 | exp wound/ or wound*.mp. [mp=title, abstract, heading word, drug trade name, original title, device manufacturer, drug manufacturer, device trade name, keyword heading word, floating subheading word, candidate term word] | 532101 |
| 11 | exp wound care/ or exp wound assessment/ or exp wound healing/ | 287377 |
| 12 | exp wound infection/ or (surgical site infection* or SSI*).mp. | 86115 |
| 13 | 10 or 11 or 12 | 592651 |
| 14 | exp image processing/ or exp image analysis/ or imag*.mp. | 2857785 |
| 15 | exp photography/ or exp videorecording/ or (photo* or video*).mp. | 1305986 |
| 16 | 14 or 15 | 3885948 |
| 17 | 5 and 9 and 13 and 16 | 274 |
| 18 | remove duplicates from 17 | 268 |

**CENTRAL**

| EBM Reviews - Cochrane Central Register of Controlled Trials <November 2023> | | |
| --- | --- | --- |
|  |  |  |
| 1 | (machine learning or artificial intelligence or AI).mp. or Artificial Intelligence/ or exp Machine Learning/ or exp Expert Systems/ or exp Pattern Recognition, Automated/ | 7931 |
| 2 | exp Neural Networks, Computer/ or (neural network* or deep learning).mp. | 2483 |
| 3 | (support vector machine* or SVM or decision tree* or gradient boost* or random forest*).mp. | 2119 |
| 4 | (computer vision or machine vision).mp. | 157 |
| 5 | 1 or 2 or 3 or 4 | 10814 |
| 6 | exp Perioperative Medicine/ or exp Perioperative Care/ or exp Perioperative Period/ or (periop* or peri-op*).mp. | 48092 |
| 7 | exp Postoperative Complications/ or (postop* or post-op*).mp. | 170641 |
| 8 | (post-surg* or post-discharge).mp. | 9647 |
| 9 | 6 or 7 or 8 | 189843 |
| 10 | wound*.mp. or exp "Wounds and Injuries"/ | 65647 |
| 11 | exp Wound Healing/ | 7594 |
| 12 | exp Wound Infection/ or (surgical site infection* or SSI*).mp. | 7721 |
| 13 | 10 or 11 or 12 | 68243 |
| 14 | exp Image Processing, Computer-Assisted/ or exp Image Interpretation, Computer-Assisted/ or imag*.mp. | 117236 |
| 15 | exp Photography/ or exp Video Recording/ or (photo* or video*).mp. | 74802 |
| 16 | 14 or 15 | 173775 |
| 17 | 5 and 9 and 13 and 16 | 14 |
| 18 | remove duplicates from 17 | 13 |

**IEEE Xplore**

(("Full Text & Metadata":"machine learning" OR "Full Text & Metadata":"artificial intelligence" OR "Full Text & Metadata":AI OR "Full Text & Metadata":"deep learning" OR "Full Text & Metadata":"neural network" OR "Full Text & Metadata":"neural networks" OR "Full Text & Metadata":"support vector machine" OR "Full Text & Metadata":"support vector machines" OR "Full Text & Metadata":SVM OR "Full Text & Metadata":"random forest" OR "Full Text & Metadata":"random forests" OR "Full Text & Metadata":"decision tree" OR "Full Text & Metadata":"decision trees" OR "Full Text & Metadata":"gradient boost" OR "Full Text & Metadata":"gradient boosting" OR "Full Text & Metadata":"gradient boosted" OR "Full Text & Metadata":"computer vision" OR "Full Text & Metadata":"machine vision") AND ("Full Text & Metadata":postop* OR "Full Text & Metadata":periop* OR "Full Text & Metadata":"post-operative" OR "Full Text & Metadata":"post-operatively" OR "Full Text & Metadata":"peri-operative" OR "Full Text & Metadata":"peri-operatively" OR "Full Text & Metadata":"post-discharge" OR "Full Text & Metadata":"post-surgery" OR "Full Text & Metadata":"post-surgical") AND ("Full Text & Metadata":wound* OR "Full Text & Metadata":“surgical site infection*” OR "Full Text & Metadata":SSI*) AND ("Full Text & Metadata":imag* OR "Full Text & Metadata":photo* OR "Full Text & Metadata":video*))

**Compendex (Engineering Village)**

Compendex for 1884-2023: (((((({Artificial intelligence} WN CV) OR ({Machine learning} WN CV) OR ({Deep learning} WN CV) OR ({Random forests} WN CV) OR ({Machine Perception} WN CV) OR ({Computer vision} WN CV) OR ({Image processing} WN CV) OR ({Pattern recognition} WN CV) OR ({Neural networks} WN CV) OR ({Expert systems} WN CV)))) OR "machine learning" OR "artificial intelligence" OR AI OR "support vector machine" OR "random forest*" OR "decision tree*" OR "gradient boost*" OR "computer vision" OR "machine vision") AND (postop* OR periop* OR post-op* OR peri-op* OR post-discharge OR post-surg*) AND (wound* OR "surgical site infection*" OR SSI*) AND ((((({Image analysis} WN CV) OR ({Image processing} WN CV) OR ({Medical image processing} WN CV) OR ({Photography} WN CV) OR ({Video recording} WN CV)))) OR imag* or photo* OR video*))

**Web of Science**

(ALL=("machine learning" OR "artificial intelligence" OR AI OR "deep learning" OR "neural network*" OR "support vector machine*" OR SVM OR "random forest*" OR "decision tree*" OR "gradient boost*" OR "computer vision" OR "machine vision")) AND ALL=((postop* OR periop* OR post-op* OR peri-op* OR post-discharge OR post-surg*)) AND ALL=((wound* OR “surgical site infection*” OR SSI*)) AND ALL=((imag* OR photo* OR video*))

**CINAHL**

| **#** | **Query** |
| --- | --- |
| S1 | (MH "Machine Learning+") OR "machine learning" OR (MH "Support Vector Machine") OR (MH "Deep Learning") OR (MH "Neural Networks (Computer)") OR (MH "Artificial Intelligence+") OR "AI" |
| S2 | "neural network*" OR "SVM*" OR "support vector machine*" |
| S3 | (MH "Random Forest") OR "random forest*" OR "decision tree*" |
| S4 | "gradient boost*" |
| S5 | "computer vision" OR "naive Bayes" OR "machine vision" |
| S6 | "deep learning" |
| S7 | (MH "Expert Systems"+) |
| S8 | S1 OR S2 OR S3 OR S4 OR S5 OR S6 OR S7 |
| S9 | (MH "Postoperative Pain") OR (MH "Postoperative Complications+") OR (MH "Postoperative Period") OR (MH "Perioperative Medicine") OR (MH "Perioperative Nursing") OR (MH "Perioperative Care+") OR (MH "Perioperative Care (Iowa NIC)+") OR (MH "Postoperative Care+") OR "postoperative" OR "perioperative" |
| S10 | "post-surg*" |
| S11 | (MH "After Care") OR "post-discharge" |
| S12 | S9 OR S10 OR S11 |
| S13 | (MH "Wound Assessment+") OR (MH "Surgical Wound Infection") OR (MH "Wound Measurement") OR (MH "Surgical Wound Dehiscence") OR (MH "Surgical Wound") OR "wound*" OR (MH "Wound Infection+") OR (MH "Wounds and Injuries+") OR (MH "Surgical Wound Care+") OR (MH "Wound Care (Saba CCC)+") OR (MH "Wound Care+") OR (MH "Wound Care (Iowa NIC)+") |
| S14 | "SSI*" OR "dehiscence" OR "surgical site infection*" |
| S15 | S13 OR S14 |
| S16 | S8 AND S12 AND S15 |
| S17 | (MH "Image Processing, Computer Assisted+") OR (MH "Image Interpretation, Computer Assisted+") |
| S18 | (MH "Photography+") |
| S19 | (MH "Videorecording+") |
| S20 | "imag*" OR "photo*" OR "video*" |
| S21 | S17 OR S18 OR S19 OR S20 |
| S22 | S16 AND S21 |

**arXiv**

("machine learning" OR "artificial intelligence" OR ai OR "deep learning" OR "neural network*" OR "support vector machine*" OR svm OR "random forest*" OR "decision tree*" OR "gradient boost*" OR "computer vision" OR "machine vision") AND (postop* OR periop* OR post-op* OR peri-op* OR post-discharge OR post-surg*) AND (wound* OR "surgical site infection*" OR ssi*) AND (imag* OR photo* OR video*)
