# Supplementary material for: Machine Learning Approaches for the Image-Based Identification of Surgical Wound Infections: Scoping Review
Source: J Med Internet Res. 2024 Jan 18;26:e52880. doi: 10.2196/52880 (PMC10835585; doi:10.2196/52880)
Supplement: Multimedia Appendix 2 [file jmir_v26i1e52880_app2.docx]

# Multimedia Appendix 2. Abstract screening tool.

1. Does the abstract indicate that humans were studied?
   1. Yes or Unsure/Unclear: continue screening
   2. No: stop screening
2. Does the abstract use English?
   1. Yes or Unsure/Unclear: continue screening
   2. No: stop screening
3. Does the abstract indicate that the record is a primary source?
   1. Yes or Unsure/Unclear: continue screening
   2. No: stop screening
4. Does the abstract indicate that surgical wounds were studied?
   1. Yes or Unsure/Unclear: continue screening
   2. No: stop screening
5. Does the abstract indicate that image-based data were used to study the surgical wounds?
   1. Yes or Unsure/Unclear: continue screening
   2. No: stop screening
6. Does the abstract indicate that a machine learning or artificial intelligence method was used for a computer vision task?
   1. Yes or Unsure/Unclear: continue screening
   2. No: stop screening
7. Does the abstract indicate that the computer vision task was identification of surgical wound infections?
   1. Yes or Unsure/Unclear: continue screening
   2. No: stop screening
