# Supplementary material for: Machine Learning Approaches for the Image-Based Identification of Surgical Wound Infections: Scoping Review
Source: J Med Internet Res. 2024 Jan 18;26:e52880. doi: 10.2196/52880 (PMC10835585; doi:10.2196/52880)
Supplement: Multimedia Appendix 3 [file jmir_v26i1e52880_app3.docx]

# Multimedia Appendix 3. Data extraction tool.

| Author(s) | e.g., Smith et al. |
| --- | --- |
| Year of publication | e.g., 2020 |
| Objective(s) | Provide a brief description of the objective of the study. |
| Source of data | Where was the data selected from (e.g., cohort, RCT, registry)? |
| Sample characteristics | How were participants recruited (e.g., inclusion and exclusion criteria)?  How many participants and/or images were there in total?  How many participants and/or images had an SSI?  What are the demographic characteristics of the included participants? |
| Postoperative context | What surgical procedure produced the wound? |
| Imaging modality | How were the images of the wound captured?  What type of image was generated?  What image preprocessing steps were taken? |
| Predictors | What candidate predictors were used?  How were predictors defined and assessed?  How were predictors selected?  How long after surgery was the wound imaged? |
| Outcome determination | How was SSI defined?  How were the SSIs identified (i.e., what reference standard was used)?  How long before/after wound imaging did SSI evaluation occur? |
| Type of study | Development, validation, or both (as per PROBAST guidelines) |
| Model development | How many models were developed?  What modeling method is used (e.g., neural network)?  How was overfitting accounted for?  How was class imbalance addressed? |
| Model evaluation | What was the method used for evaluating model performance (e.g., cross-validation, external validation)? |
| Model performance | What are the calibration and classification measures that are reported? |
| Key findings | Note how the results were interpreted by the authors. |
| Limitations | Note any limitations described by the authors. |
