# Supplementary material for: Machine Learning Approaches for the Image-Based Identification of Surgical Wound Infections: Scoping Review
Source: J Med Internet Res. 2024 Jan 18;26:e52880. doi: 10.2196/52880 (PMC10835585; doi:10.2196/52880)
Supplement: Multimedia Appendix 4 [file jmir_v26i1e52880_app4.docx]

**Multimedia Appendix 4.** TRIPOD and PROBAST altered or excluded items.

**TRIPOD items**

| **TRIPOD item** | | **Alteration for this study** |
| --- | --- | --- |
| 2vii | Predictors included in the final model are reported in the abstract | Not applicable to DL studies (the number of images is proportional to the number of participants (which is addressed in items 2iv and v)). For non-DL studies, features included in the final model should be mentioned. |
| 6aiii | It is described when the outcome was assessed (time point(s) since T0) | Not applicable for studies in which outcome assessment was based solely on images, as the time of outcome assessment is practically the same as the time of image capture (item 7aiv). |
| 6b | Report any actions to blind assessment of the outcome to be predicted. | Not applicable to image-based studies. Assessment of wound infection is largely visual; visual assessment cannot be meaningfully blinded to the image of the wound. |
| 7ai | All predictors are reported | Not applicable to DL studies, as predictors are automatically generated from images without human input. Non-DL studies (using hand-crafted features) should report (type of) features used (e.g., local Gabor wavelet binary pattern histograms). |
| 7aii | Predictor definitions are clearly presented | Not applicable to DL studies, as predictors are automatically generated from images without human input. Non-DL studies (using hand-crafted features) should report how features were derived from the images (e.g., ‘computed by dividing the image into 3×3 blocks and…’). |
| 7aiii | It is clearly described how the predictors were measured | For all studies, interpreted as ‘It is clearly described how the images were taken’. Both the device and person(s) responsible should be reported. |
| 7aiv | It is clearly described when the predictors were measured | For all studies, interpreted as ‘It is clearly described when the images were taken’. |
| 7bi | It is clearly described whether predictor assessments were blinded for outcome | Interpreted as 'It is clearly described whether any actions to standardize image capture or otherwise systematically capture the wound from the image were taken’. |
| 7bii | It is clearly described whether predictor assessments were blinded for the other predictors | Not assessed as there is no human-assessed predictor provided to the model |
| 9 | Describe how missing data were handled (e.g., complete-case analysis, single imputation, multiple imputation) with details of any imputation method. | Not applicable as missing images can only be handled by complete-case analysis and cannot be imputed. |
| 10a | Describe how predictors were handled in the analyses. | Not applicable to DL studies, as predictors are automatically generated from images without human input |
| 13a | Describe the flow of participants through the study, including the number of participants with and without the outcome and, if applicable, a summary of the follow-up time. | Flow of images rather than patients can be reported. |
| 13bii | Summary information is provided for all predictors included in the final developed/validated model | For DL studies, we searched for any interpretable visualizations of important regions of the image used in the prediction |
| 13biii | The number of participants with missing data for predictors is reported | Not assessed as missing images can only be handled by complete-case analysis and cannot be imputed. |
| 13biv | The number of participants with missing data for the outcome is reported | Not assessed as missing images can only be handled by complete-case analysis and cannot be imputed. |
| 14ai | The number of participants in each analysis (e.g. in the analysis of each model if more than one model is developed) is specified | Interpreted as ‘The numbers of participants/images in training and testing sets are specified’ |
| 14aii | The number of outcome events in each analysis is specified (e.g. in the analysis of each model if more than one model is developed) | Interpreted ‘The numbers of participants/images with wound infection in training and testing sets are specified’ |
| 15a | Present the full prediction model to allow predictions for individuals (i.e., all regression coefficients, and model intercept or baseline survival at a given time point). | The prediction model should be made available to readers (e.g., by providing code). For studies using regression-based approaches, this item applies as written. |

**PROBAST items**

| **PROBAST item** | | **Alteration for this study** |
| --- | --- | --- |
| 2.1 | Were predictors defined and assessed in a similar way for all participants? | Interpreted as ‘Were images taken in a pre-defined/consistent way or were wounds otherwise systematically captured from the image?’ |
| 2.2 | Were predictor assessments made without knowledge of outcome data? | Not assessed as there is no human-assessed predictor provided to the model (all features directly computed from image provided to model). |
| 2.3 | Are all predictors available at the time the model is intended to be used? | Not assessed as predictors can be computed for any image. |
| 3.3 | Were predictors excluded from the outcome definition? | Not assessed as the definition of wound infection includes visual criteria; predictors are derived from the same visual information. |
| 3.5 | Was the outcome determined without knowledge of predictor information? | Not assessed as assessment of wound infection is largely visual; visual assessment cannot be meaningfully blinded to the image of the wound. |
| 4.1 | Were there a reasonable number of participants with the outcome? | With developments in transfer learning, the authors are looking for some degree of justification/reasoning for DL studies with respect to the number of participants/images studied. If TL is not used (or non-DL studies), similarly, we are looking for some degree of justification of the number of images used. |
| 4.2 | Were continuous and categorical predictors handled appropriately? | Not applicable to DL studies, predictors are automatically generated from images without human input. |
| 4.4 | Were participants with missing data handled appropriately? | Not applicable as missing images can only be handled by complete-case analysis and cannot be imputed |
| 4.5 | Was selection of predictors based on univariable analysis avoided? | Not applicable to DL studies, predictors are automatically generated and selected from images without human input |
| 4.6 | Were complexities in the data (e.g. censoring, competing risks, sampling of controls) accounted for appropriately? | Rule of thumb: one class being 10% or less indicates high risk of bias |
| 4.9 | Do predictors and their assigned weights in the final model correspond to the results from multivariable analysis? | Not applicable to DL or SVM studies as predictors and assigned weights are not available as closed-form equations.  For studies using regression-based approaches, this item applies as written. |

Applicability was not assessed since the review’s eligibility criteria were quite conservative and ensured that included studies were relevant to the review questions and objectives.

**Additional questions added to PROBAST**

*Domain 4: Analysis*

Is all data from a single patient reserved to only a single data partition (e.g., training, testing or tuning)?
