# Supplementary material for: Machine Learning Approaches for the Image-Based Identification of Surgical Wound Infections: Scoping Review
Source: J Med Internet Res. 2024 Jan 18;26:e52880. doi: 10.2196/52880 (PMC10835585; doi:10.2196/52880)
Supplement: Multimedia Appendix 5 [file jmir_v26i1e52880_app5.docx]

**Table S1. Complete data extraction table.**

| **Title** | **Author(s)** | **Year** | **Objective(s)** | **Source of data** |
| --- | --- | --- | --- | --- |
| The Use of Mobile Thermal Imaging and Deep Learning for Prediction of Surgical Site Infection | Fletcher et al. | 2021 | To develop a model for predicting SSI in C-section wounds from thermal images taken with smartphones. | Cross-sectional cohort |
| Use of Convolutional Neural Nets and Transfer Learning for Prediction of Surgical Site Infection from Color Images. | Fletcher et al. | 2021 | To develop a model for predicting SSI in C-section wounds from colour images taken with mobile devices. | Cross-sectional cohort |
| Application of Machine Learning to Prediction of Surgical Site Infection | Fletcher et al. | 2019 | To develop a model for predicting SSI in C-section wounds from questionnaire and image data. | Cross-sectional cohort |
| A Unified Framework for Automatic Detection of Wound Infection with Artificial Intelligence | Wu et al. | 2020 | To develop an automatic monitoring tool for surgical wounds based on smartphone images. | Prospective cohort |
| Deepwound: Automated Postoperative Wound Assessment and Surgical Site Surveillance through Convolutional Neural Networks | Shenoy et al. | 2018 | To develop a model that can identify the onset of wound ailments from smartphone images. | Not reported |
| Automatic Wound Infection Interpretation for Postoperative Wound Image | Hsu et al. | 2017 | To develop a model for recognizing surgical site infection. | Retrospective cohort |
| Chronic wound assessment and infection detection method. | Hsu et al. | 2019 | To develop an automatic wound interpretation app for automated wound monitoring. | Retrospective cohort |
| Implementation of Post-operative Wound Analytics | Zeng et al. | 2017 | To develop a system for automatic wound detection and subsequent infection detection. | Not reported |
| Photographic LVAD Driveline Wound Infection Recognition Using Deep Learning. | Luneburg et al. | 2019 | To explore machine learning approaches for driveline wound infection recognition from photographs alone. | Unclear, potentially retrospective |
| A unified framework for automatic wound segmentation and analysis with deep convolutional neural networks | Wang et al. | 2015 | To develop an integrated system for automatic wound segmentation and analysis of wound conditions from wound images. | Unclear, potentially retrospective |

| **Sample characteristics** | | | | |
| --- | --- | --- | --- | --- |
| **Recruitment method & criteria** | **Number and location of sites** | **Demographics** | **Total number of participants and/or images** | **Number of participants and/or images with SSI** |
| All women who underwent C-section at a particular hospital in Kigali, Rwanda between Sep 2019 and Feb 2020 were prospectively enrolled on POD1 | One site: Kirehe District Hospital in Kigali, Rwanda | No additional information reported | 530 participants | 30 participants |
| Women aged 18+ who underwent C-section at a particular hospital in Kigali, Rwanda between Mar and Oct 2017 were enrolled prior to discharge | One site: Kirehe District Hospital in Kigali, Rwanda | No additional information reported | Out of 729 eligible participants, 572 returned for follow-up | 62 participants |
| Women aged 18+ who underwent C-section at a particular hospital in Kigali, Rwanda between Mar and Oct 2017 were enrolled prior to discharge | One site: Kirehe District Hospital in Kigali, Rwanda | No additional information reported | Out of 729 eligible participants, 572 returned for follow-up; images from 568 patients were available | Unclear, 61 or 62 participants |
| Patients undergoing surgery at "an Asian academic center", specific criteria not reported though surgical procedures are indicated | Specific site not specified | Not reported | 480 wound images from 100 patients | 136 images |
| Not reported | "primarily" from two sites: Palo Alto VA Hospital and the Washington University Medical Center in St. Louis | Not reported | 1335 images | 355 images |
| Not reported | One site: Department of Surgery at the National Taiwan University Hospital | Not reported | 42 wound images | 30 images |
| Not reported | Two departments at one site: Department of Surgery and Department of Internal Medicine of National Taiwan University Hospital | Not reported | 293 wound images | Training: 27 infection images |
| Not reported | Not reported | Not reported | Total unclear; 6 images for testing | Not reported |
| Not reported | Two sites: Schüchtermann-Schiller’sche Kliniken and Hannover Medical School | Not reported | 745 images from 61 patients, though only 732 are labelled. Only 100 images are used for the 'blind evaluation' of infection classification. | 212 mild infection, 37 severe infection.  In 'blind evaluation' dataset: 27 mild infection, 15 severe infection. |
| Not reported | NYU | Not reported | 3400 images | 155 images |

**Table S1. (cont.)**

**Table S1. (cont.)**

| **Postoperative context** | **Imaging modality** | | |
| --- | --- | --- | --- |
| **Surgical procedure(s)** | **Type of image** | **Image pre-processing** | **Image collection** |
| C-section | Thermal camera module connected to smartphone that produces JPG thermal image and separate 2D temperature array | 2D temperature array used to create a thermal pseudo-image, but unclear if these are used.   CV script used to find outline of each wound frame and crop thermal images to 160×100. | It seems like a "health worker" took the images (based on Fig. 1). Unclear how many different individuals took the images. |
| C-section | Colour image taken with Android tablet | For TL model: image size reduced to 224×224 | Images taken by designated community health workers. Unclear how many different individuals took the images. |
| C-section | Colour image taken with Android tablet | Images cropped to only include skin. Scaled to 160×340. Converted from RGB to CIELAB. | Images taken by designated community health workers.  "No specific guidelines were given for capturing the images". Unclear how many different individuals took the images. |
| Laparatomy, minimal invasive surgery, or hernia repair | Colour image taken with smartphone | Wound seam, peri-wound area, and region of interest were annotated prior to annotation of infection features for model to locate wounds. Log transform followed by gamma transform for brightness correction.  Color correction using a correction matrix based on a CameraTrax color card. | Images taken by surgeons |
| Not reported | Colour image taken with smartphone | Contrast-limited adaptive histogram equalization (CLAHE) applied to each image to accommodate differences in lighting/position. | Images taken primarily from patients and surgeons |
| Not reported | Colour image; device not reported | After segmentation, the wound area is automatically detected through suture site recognition, for which the images are converted to greyscale. This is followed by ROI detection via hierarchical clustering to group 'feature points'. | Not reported |
| Specific procedures not reported, but areas of the body include chest, abdomen, back, hand, and feet | Colour image taken with smartphone (iPhone 6+ and Samsung Galaxy S6) | After segmentation, the wound area is automatically detected through suture site recognition, for which the images are converted to greyscale. Otsu’s adaptive thresholding is then applied to generate a binary image. This is followed by ROI detection via hierarchical clustering to group 'feature points'. | No details provided, but images were taken "under different settings and capture conditions" |
| Not reported | Colour image; device not reported | Before infectious status recognition, the system performs automatic wound detection: Areas with "higher textural characteristics" are identified using difference of Gaussians (edge detection). This is followed by color normalization and separation of the skin color and non-skin color areas. Final wound area is determined from edge and color detection. | Not reported |
| LVAD implantation with driveline | Colour image; device not reported | Highly out-of-focus images were filtered out of the training data (using an algorithm based on the variance of the Laplacian). This was followed by driveline tube segmentation (also using a CNN) and ROI prediction. | Images taken from various positions and lighting conditions, some out of focus or showing signs of camera motion |
| Not reported | Colour image; device not reported | Images cropped to 480x640. Wound segmentation was performed using a convolutional encoder-decoder network that produces segmentation masks. However, it seems like infection classification was performed on unsegmented image. | Not reported |

**Table S1. (cont.)**

| **Predictors** | | |
| --- | --- | --- |
| **Candidate predictors** | **Predictor definition & assessment** | **Time between surgery and wound imaging** |
| Unclear (DL) | N/A | approximately 10 days after surgery |
| Unclear (DL) | N/A | approximately 10 days after surgery |
| Texture features from Gabor wavelet analysis and colour features from statistical colour analysis. Total number of initial features is unclear, but seems to be 7784 | For texture features, the image was divided into 9 (3x3) blocks; Gabor wavelet analysis applied to each block to obtain local Gabor binary pattern histograms. For colour features, the image was divided into 8 (4x2 blocks); mean, variance and skewness of the pixel values were computed per block. | approximately 10 days after surgery |
| Unclear (DL) For non-DL models, predictors are also unclear | N/A | Images taken just after surgery, during hospitalization, and in outpatient clinic follow-up (number of days not reported) |
| Unclear (DL) | N/A | Not reported |
| Different feature vectors for each of the four modules (swelling, blood region, infection, necrosis) | Definition/steps only provided for Swelling (the Swelling feature vector was calculated by taking into account pixel-level hue histograms) | Not reported |
| Different feature vectors for each of the four modules (swelling, granulation, infection, necrosis) | Definition/steps only provided for Swelling (the Swelling feature vector was calculated by taking into account pixel-level hue histograms) | Not reported |
| Six histogram-based features; 20 GLCM-based features with 4 orientations and 1 distance. Total of 258 features. | Color-normalized image divided into h×w-sized blocks; features are computed per block and per color channel. (SVM only analyzes blocks that overlap with the wound area). | Not reported |
| Unclear (DL) | N/A | Not reported |
| Unclear (DL). "hidden layer activations calculated by the ConvNet were used as our features". Feature dimension reduced by a factor of 25 via spatial pooling. | N/A | Not reported |

| **Outcome determination** | | | | **Type of study** |
| --- | --- | --- | --- | --- |
| **Outcome(s) of interest** | **SSI assessment** | **SSI definition** | **Time between wound imaging and SSI assessment** |  |
| SSI | Performed by a general practitioner (physical exam). Unclear how many different GPs. | No specific criteria reported (expert opinion) | SSI assessment occurred on POD11 (+/- 3 days), "in conjunction with" thermal imaging | D |
| SSI | Unclear -- abstract indicates physical exam; main text indicates examination of the images | No specific criteria reported (expert opinion) | Likely on the same day as imaging | D |
| SSI | Performed by a general practitioner (physical exam). Unclear how many different GPs. | No specific criteria reported (expert opinion) | Likely on the same day as imaging | D |
| SSI | Surgeons annotated abnormal wound features (e.g., necrosis, pus) on the images. | Wound infection was defined as presence of redness, pus, or necrosis. | Separate SSI assessment did not occur (only annotation of images) | D |
| Wound presence, infection (SSI), granulation tissue, drainage, fibrinous exudate, open wound, staples, steri strips, sutures | Not reported | Not reported | Not reported | D |
| Swelling, 'blood region', infection, necrosis | Unclear, potentially performed by surgeons | Unclear, potentially based on expert opinion | Not reported | D |
| Swelling, granulation, infection, tissue necrosis | Unclear, there is manual annotation of images, potentially based on majority vote of three physicians | No specific criteria reported (expert opinion) | Separate SSI assessment did not occur (only annotation of images) | D |
| three "infectious statuses" (blood region, swelling, and necrosis) and 'normal' status | Not reported | Not reported | Not reported | D |
| Driveline infection (none, mild, or severe) | Not entirely clear, though it seems like assessment was performed by "clinical experts" based on physical exam | Based on features such as presence of bacteria, odour and warmth, in addition to visual features on the surface of the wound; specific criteria not reported | Not reported | D |
| SSI | Unclear, but authors "created … binary infection labels" | Not reported | Not reported | D |

**Table S1. (cont.)**

| **Model development** | | | | |
| --- | --- | --- | --- | --- |
| **Modeling methods** | **Number of infection detection models developed** | **Classification task** | **Overfitting** | **Class imbalance** |
| CNN | Two: a naïve few-layer CNN and a ResNet50-initiated model | Binary | Data augmentation (random image rotation, image flip), batch normalization, and node dropout (50% for naïve, 20% for TL) | data synthesis (limited to a factor of 2); class weights adjusted (relative weights ranging from 5 to 15; optimal ratio was 1:15) |
| CNN | Two: a naïve few-layer CNN and a ResNet50-initiated model | Binary | Data augmentation (random image rotation, image flip), batch normalization, and node dropout (50% for naïve, 20% for TL) | SMOTE (amount of data synthesis limited to a factor of 2); class weights adjusted (relative weights ranging from 0 to 20; optimal ratio was 1:9); use of log loss as loss function |
| Unclear; potentially both SVM and L1-penalized logistic regression | Unclear, potentially three image-based models but only results for one are reported in main text. There are additional questionnaire-based models | Binary | Not reported | Not reported |
| CNN, SVM, RF, GB | Four | Binary | Not reported | Not reported |
| CNN | Three VGG-16-initialized models with ImageNet weights. A different layer is frozen for each model. These three are used to create a single model (ensemble) | Multilabel | Ensemble learning. Data augmentation strategies include random rotation, 10-pixel shifts in any direction, 30% zoom, 20% shearing, vertical & horizontal flipping. | Not reported |
| SVM | One | Multiclass | Not reported | Not reported |
| SVM | One | Multiclass | Not reported | Not reported |
| SVM | One with linear kernel, one with RBF kernel | Unclear, but likely multiclass | Not reported | Not reported |
| CNN | Unclear, it seems like multiple architectures were tested but the number is not reported. Results are presumably reported for the best performing architecture, which was VGG-16 pretrained on ImageNet | Multiclass | Data augmentation using affine transformations (though authors do not mention overfitting explicitly) | Accuracy was weighted by class distribution |
| SVM using CNN features | One with linear kernel, one with polynomial kernel | Binary | Not reported | Not reported |

**Table S1. (cont.)**

**Table S1. (cont.)**

| **Method for model evaluation** | **Model performance** | |
| --- | --- | --- |
|  | Calibration | Discrimination & Classification |
| 5- and 10-fold cross-validation | Not reported | Naïve: median AUC = 0.85; sensitivity = 0.71; specificity = 0.87; MCC = 0.37.   TL:  median AUC = 0.90; sensitivity = 0.95; specificity = 0.84; MCC = 0.44 |
| 5- and 10-fold cross-validation | Not reported | Naïve: median AUC = 0.655; sensitivity = 0.75; specificity = 0.58  TL:  median AUC = 0.639; sensitivity = 0.93; specificity = 0.18 |
| 100 randomly generated splits of the data | Not reported | Unclear, but probably:  AUC = 1.0; sensitivity = 1.0; specificity = 1.0 |
| 5-fold cross-validation | Not reported | CNN: AUC = 0.833; recall = 0.771; specificity = 0.822  SVM: AUC = 0.444; recall = 0.408; specificity = 0.562  RF: AUC = 0.671; recall = 0.648; specificity = 0.192  GB: AUC = 0.669; recall = 0.652; specificity = 0.652 |
| Train-test split | Not reported | (Ensemble model results)For infection detection: accuracy = 0.84; sensitivity = 0.70; specificity = 0.70; F1 score = 0.70; AUC = 0.82 |
| Unclear, potentially cross-validation | Not reported | "wound infection interpretation achieved 95.23% accuracy, 93.33% sensitivity, 100% specificity, and 100% PPV". Unclear if this is specific to the infection module or if it encompasses all four modules |
| Unclear, potentially cross-validation | Not reported | Symptom Assessment: accuracy = 0.8358; 189 TPs, 259 TNs, 24 FPs, 64 FNs. For wound infection module specifically: accuracy = 0.8582; 44 TPs, 71 TNs, 2 FPs, 17 FNs. |
| 10-fold cross validation | Not reported | Linear kernel Normal: AUC = 0.9145; sensitivity = 0.8935;  specificity = 0.7250; accuracy = 0.8590 Blood region: AUC = 0.8050; sensitivity = 0.6222;  specificity = 0.8138; accuracy = 0.7684 Swelling: AUC = 0.8542; sensitivity = 0.6545; specificity = 0.8519; accuracy = 0.7947 Necrosis: AUC = 0.7640; sensitivity = 0.5667;  specificity = 0.8241; accuracy = 0.7632  RBF kernel: Normal: AUC = 0.8298; sensitivity = 0.9161;  specificity = 0.7250; accuracy = 0.8769 Blood region: AUC = 0.8533; sensitivity = 0.6889;  specificity = 0.9310; accuracy = 0.8737 Swelling: AUC = 0.8471; sensitivity = 0.4182;  specificity = 0.9037; accuracy = 0.7632 Necrosis: AUC = 0.7682; sensitivity = 0.4878;  specificity = 0.8931; accuracy = 0.7947 |
| Leave-one-out cross-validation | Not reported | No infection: accuracy = 0.810; F1 = 0.80 Mild infection: accuracy = 0.667; F1 = 0.57 Severe infection: accuracy = 0.133; F1 = 0.20 Total / macro: accuracy = 0.670; F1 = 0.52 |
| 5-fold cross-validation | Not reported | Linear kernel: AUC = 0.763; recall = 0.231; precision = 0.333; F1 = 0.273; accuracy = 0.953  Polynomial kernel: AUC = 0.847; recall = 0.308; precision = 0.400; F1 = 0.348; accuracy = 0.956 |

**Table S1. (cont.)**

| **Key findings** | **Limitations** |
| --- | --- |
| Successful prediction of SSI using thermal images alone. | Class imbalance; additional testing with larger dataset required |
| Modest ability to predict SSI from colour images. | AUC is moderate. Further data needed to test generalizability. Ambient lighting and skin color may affect model performance. |
| Successful prediction of SSI from colour images. | The questionnaire-based model's performance may be limited to due inaccurate answers. Homogeneity in skin colour may limit use in other populations |
| Authors designed a system that can automatically detect wound area and recognize infection. | Potentially limited generalizability to other populations, inter-person variation in photograph quality, lack of test set, relatively small dataset |
| Accurate multilabel classification of wound images using a CNN ensemble. | Class imbalance, relatively small dataset |
| Succesful wound segmentation and infectious status recognition using an SVM | None reported in paper |
| Succesful wound segmentation and infectious status recognition using an SVM | None reported in paper |
| This is an early work that was able to develop a system for quick and automated wound detection and infectious status recognition. | None reported in paper |
| Modest ability to classify driveline wounds by infection class. The authors concluded that photographic data may not be sufficient for accurate infection classification in all cases. | None reported in paper |
| First paper to develop an automated system for wound infection detection using DL. | None reported in paper |

| **Title** | **Author(s)** | **Type of study** | **ML Method** | **Title** | | | | |
| --- | --- | --- | --- | --- | --- | --- | --- | --- |
|  |  |  |  | **1** | | | | |
|  |  |  |  | **i** | **ii** | **iii** | **iv** | **Score** |
| The Use of Mobile Thermal Imaging and Deep Learning for Prediction of Surgical Site Infection | Fletcher et al. | D | CNN | N | Y | N | Y | 0 |
| Use of Convolutional Neural Nets and Transfer Learning for Prediction of Surgical Site Infection from Color Images. | Fletcher et al. | D | CNN | N | Y | N | Y | 0 |
| Application of Machine Learning to Prediction of Surgical Site Infection | Fletcher et al. | D | Unclear; potentially SVM and L1-penalized logistic regression | N | Y | N | Y | 0 |
| A Unified Framework for Automatic Detection of Wound Infection with Artificial Intelligence | Wu et al. | D | CNN, SVM, RF, GB | N | N | N | Y | 0 |
| Deepwound: Automated Postoperative Wound Assessment and Surgical Site Surveillance through Convolutional Neural Networks | Shenoy et al. | D | CNN | N | N | N | N | 0 |
| Automatic Wound Infection Interpretation for Postoperative Wound Image | Hsu et al. | D | SVM | N | N | N | Y | 0 |
| Chronic wound assessment and infection detection method. | Hsu et al. | D | SVM | N | N | N | Y | 0 |
| Implementation of Post-operative Wound Analytics | Zeng et al. | D | SVM | N | N | N | N | 0 |
| Photographic LVAD Driveline Wound Infection Recognition Using Deep Learning. | Luneburg et al. | D | CNN | N | N | Y | Y | 0 |
| A unified framework for automatic wound segmentation and analysis with deep convolutional neural networks | Wang et al. | D | SVM using CNN features | N | N | N | N | 0 |

**Table S2. Complete assessment of TRIPOD employment.**

**Table S2. (cont.)**

| **Abstract** | | | | | | | | | | | | |
| --- | --- | --- | --- | --- | --- | --- | --- | --- | --- | --- | --- | --- |
| **2** | | | | | | | | | | | | |
| **i** | **ii** | **iii** | **iv** | **v** | **vi** | **vii** | **viii** | **ix** | **x** | **xi** | **xii** | **Score** |
| Y | Y | Y | Y | Y | Y | NA | Y | Y | Y | N | Y | 0 |
| Y | Y | Y | Y | Y | Y | NA | Y | Y | Y | N | N | 0 |
| Y | Y | Y | Y | Y | Y | N | Y | Y | Y | N | Y | 0 |
| Y | Y | Y | N | Y | N | N | Y | N | Y | N | Y | 0 |
| Y | N | Y | N | N | N | NA | Y | Y | N | N | N | 0 |
| Y | Y | N | N | Y | N | N | Y | Y | N | N | N | 0 |
| Y | Y | N | N | N | N | N | Y | Y | N | N | Y | 0 |
| Y | N | N | N | N | N | N | N | N | N | N | Y | 0 |
| Y | N | N | Y | N | N | NA | Y | Y | N | N | Y | 0 |
| Y | N | N | N | N | N | NA | Y | N | N | N | Y | 0 |

| **Background and objectives** | | | | | **Source of data** | | | | | |
| --- | --- | --- | --- | --- | --- | --- | --- | --- | --- | --- |
| **3a** | | | **3b** | | **4a** | | **4b** | | | |
| **i** | **ii** | **Score** | **i** | **Score** | **i** | **Score** | **i** | **ii** | **iii** | **Score** |
| Y | Y | 1 | Y | 1 | Y | 1 | Y | Y | NA | 1 |
| Y | Y | 1 | Y | 1 | Y | 1 | Y | Y | NA | 1 |
| Y | Y | 1 | Y | 1 | Y | 1 | Y | Y | NA | 1 |
| Y | Y | 1 | Y | 1 | Y | 1 | N | N | NA | 0 |
| Y | Y | 1 | Y | 1 | N | 0 | N | N | NA | 0 |
| Y | N | 0 | Y | 1 | Y | 1 | N | N | NA | 0 |
| Y | Y | 1 | Y | 1 | Y | 1 | N | N | NA | 0 |
| Y | Y | 1 | Y | 1 | N | 0 | N | N | NA | 0 |
| Y | N | 0 | Y | 1 | N | 0 | N | N | NA | 0 |
| Y | Y | 1 | Y | 1 | N | 0 | N | N | NA | 0 |

**Table S2. (cont.)**

| **Participants** | | | | | | | | **Outcome** | | | | | |
| --- | --- | --- | --- | --- | --- | --- | --- | --- | --- | --- | --- | --- | --- |
| **5a** | | | | **5b** | | **5c** | | **6a** | | | | **6b** | |
| **i** | **ii** | **iii** | **Score** | **i** | **Score** | **i** | **Score** | **i** | **ii** | **iii** | **Score** | **i** | **Score** |
| Y | Y | Y | 1 | Y | 1 | NA | NA | N | Y | Y | 0 | NA | NA |
| Y | Y | Y | 1 | Y | 1 | NA | NA | N | Y | Y | 0 | NA | NA |
| Y | Y | Y | 1 | Y | 1 | NA | NA | N | Y | Y | 0 | NA | NA |
| Y | Y | Y | 1 | N | 0 | NA | NA | Y | Y | NA | 1 | NA | NA |
| N | Y | Y | 0 | N | 0 | NA | NA | N | N | N | 0 | NA | NA |
| N | Y | Y | 0 | N | 0 | NA | NA | N | Y | NA | 0 | NA | NA |
| Y | Y | Y | 1 | N | 0 | NA | NA | N | Y | NA | 0 | NA | NA |
| N | N | N | 0 | N | 0 | NA | NA | N | N | N | 0 | NA | NA |
| Y | Y | Y | 1 | N | 0 | NA | NA | N | Y | N | 0 | NA | NA |
| N | Y | Y | 0 | N | 0 | NA | NA | N | N | N | 0 | NA | NA |

**Table S2. (cont.)**

| **Predictors** | | | | | | | | **Sample size** | | **Missing data** | | | | |
| --- | --- | --- | --- | --- | --- | --- | --- | --- | --- | --- | --- | --- | --- | --- |
| **7a** | | | | | **7b** | | | **8** | | **9** | | | | |
| **i** | **ii** | **iii** | **iv** | **Score** | **i** | **ii** | **Score** | **i** | **Score** | **i** | **ii** | **iii** | **iv** | **Score** |
| NA | NA | Y | Y | 1 | Y | NA | 1 | N | 0 | NA | NA | NA | NA | NA |
| NA | NA | Y | Y | 1 | Y | NA | 1 | Y | 1 | NA | NA | NA | NA | NA |
| Y | Y | Y | Y | 1 | Y | NA | 1 | Y | 1 | NA | NA | NA | NA | NA |
| N | N | Y | N | 0 | Y | NA | 1 | N | 0 | NA | NA | NA | NA | NA |
| NA | NA | Y | N | 0 | Y | NA | 1 | N | 0 | NA | NA | NA | NA | NA |
| Y | N | N | N | 0 | Y | NA | 1 | N | 0 | NA | NA | NA | NA | NA |
| Y | N | N | N | 0 | Y | NA | 1 | N | 0 | NA | NA | NA | NA | NA |
| Y | Y | N | N | 0 | N | NA | 0 | N | 0 | NA | NA | NA | NA | NA |
| NA | NA | N | N | 0 | Y | NA | 1 | N | 0 | NA | NA | NA | NA | NA |
| NA | NA | N | N | 0 | Y | NA | 1 | N | 0 | NA | NA | NA | NA | NA |

**Table S2. (cont.)**

| **Statistical analysis methods** | | | | | | | | | | | | | | | | | | |
| --- | --- | --- | --- | --- | --- | --- | --- | --- | --- | --- | --- | --- | --- | --- | --- | --- | --- | --- |
| **10a** | | | | **10b** | | | | | | | **10c** | | **10d** | | | | **10e** | |
| **i** | **ii** | **iii** | **Score** | **i** | **ii** | **iii** | **iv** | **v** | **vi** | **Score** | **i** | **Score** | **i** | **ii** | **iii** | **Score** | **i** | **Score** |
| NA | NA | NA | NA | Y | NA | NA | NA | NA | Y | 1 | NA | NA | Y | N | Y | 0 | NA | NA |
| NA | NA | NA | NA | Y | NA | NA | NA | NA | Y | 1 | NA | NA | Y | N | Y | 0 | NA | NA |
| N | NA | NA | 0 | Y | NA | Y | N | NA | Y | 0 | NA | NA | N | N | N | 0 | NA | NA |
| N | NA | NA | 0 | Y | N | N | N | NA | Y | 0 | NA | NA | Y | N | Y | 0 | NA | NA |
| NA | NA | NA | NA | Y | NA | NA | NA | NA | Y | 1 | NA | NA | N | N | N | 0 | NA | NA |
| N | NA | NA | 0 | Y | NA | N | N | NA | N | 0 | NA | NA | N | N | N | 0 | NA | NA |
| N | NA | NA | 0 | Y | NA | N | N | NA | N | 0 | NA | NA | N | N | N | 0 | NA | NA |
| N | NA | NA | 0 | Y | NA | N | N | NA | Y | 0 | NA | NA | N | N | N | 0 | NA | NA |
| NA | NA | NA | NA | Y | NA | NA | NA | NA | Y | 1 | NA | NA | N | N | N | 0 | NA | NA |
| NA | NA | NA | NA | Y | NA | N | N | NA | Y | 0 | NA | NA | N | N | N | 0 | NA | NA |

**Table S2. (cont.)**

| **Risk groups** | | **Development vs. validation** | | **Participants** | | | | | | | | | | | | |
| --- | --- | --- | --- | --- | --- | --- | --- | --- | --- | --- | --- | --- | --- | --- | --- | --- |
| **11** | | **12** | | **13a** | | | | **13b** | | | | | **13c** | | | |
| **i** | **Score** | **i** | **Score** | **i** | **ii** | **iii** | **Score** | **i** | **ii** | **iii** | **iv** | **Score** | **i** | **ii** | **iii** | **Score** |
| NA | NA | NA | NA | N | Y | NA | 0 | N | N | NA | NA | 0 | NA | NA | NA | NA |
| NA | NA | NA | NA | Y | Y | NA | 1 | N | N | NA | NA | 0 | NA | NA | NA | NA |
| NA | NA | NA | NA | Y | Y | NA | 1 | N | N | NA | NA | 0 | NA | NA | NA | NA |
| NA | NA | NA | NA | N | Y | NA | 0 | N | N | NA | NA | 0 | NA | NA | NA | NA |
| NA | NA | NA | NA | N | Y | NA | 0 | N | Y | NA | NA | 0 |  |  |  |  |
| NA | NA | NA | NA | N | Y | NA | 0 | N | N | NA | NA | 0 | NA | NA | NA | NA |
| NA | NA | NA | NA | N | Y | NA | 0 | N | N | NA | NA | 0 |  |  |  |  |
| NA | NA | NA | NA | N | N | NA | 0 | N | N | NA | NA | 0 | NA | NA | NA | NA |
| NA | NA | NA | NA | N | Y | NA | 0 | N | N | NA | NA | 0 |  |  |  |  |
| NA | NA | NA | NA | N | Y | NA | 0 | N | N | NA | NA | 0 | NA | NA | NA | NA |

**Table S2. (cont.)**

**Table S2. (cont.)**

| **Model development** | | | | | **Model specification** | | | | |
| --- | --- | --- | --- | --- | --- | --- | --- | --- | --- |
| **14a** | | | **14b** | | **15a** | | | **15b** | |
| **i** | **ii** | **Score** | **i** | **Score** | **i** | **ii** | **Score** | **i** | **Score** |
| Y | Y | 1 | NA | NA | N | NA | 0 | N | 0 |
| Y | Y | 1 | NA | NA | N | NA | 0 | N | 0 |
| Y | Y | 1 | NA | NA | N | NA | 0 | N | 0 |
| Y | Y | 1 | NA | NA | N | NA | 0 | Y | 1 |
| Y | N | 0 | NA | NA | N | NA | 0 | Y | 1 |
| Y | N | 0 | NA | NA | N | NA | 0 | N | 0 |
| Y | N | 0 | NA | NA | N | NA | 0 | Y | 1 |
| N | N | 0 | NA | NA | N | NA | 0 | N | 0 |
| Y | Y | 1 | NA | NA | N | NA | 0 | N | 0 |
| Y | Y | 1 | NA | NA | N | NA | 0 | N | 0 |

| **Model performance** | | | | | **Model updating** | | | | | |
| --- | --- | --- | --- | --- | --- | --- | --- | --- | --- | --- |
| **16** | | | | | **17** | | | | | |
| **i** | **ii** | **iii** | **iv** | **Score** | **i** | **ii** | **iii** | **iv** | **v** | **Score** |
| Y | N | N | Y | 0 | NA | NA | NA | NA | NA | NA |
| Y | N | N | Y | 0 | NA | NA | NA | NA | NA | NA |
| Y | N | N | Y | 0 | NA | NA | NA | NA | NA | NA |
| Y | N | N | Y | 0 | NA | NA | NA | NA | NA | NA |
| Y | N | N | Y | 0 | NA | NA | NA | NA | NA | NA |
| N | N | N | Y | 0 | NA | NA | NA | NA | NA | NA |
| N | N | N | Y | 0 | NA | NA | NA | NA | NA | NA |
| Y | N | N | Y | 0 | NA | NA | NA | NA | NA | NA |
| N | N | N | Y | 0 | NA | NA | NA | NA | NA | NA |
| Y | N | N | Y | 0 | NA | NA | NA | NA | NA | NA |

**Table S2. (cont.)**

| **Limitations** | | **Interpretation** | | | | **Implications** | | | **Supplementary information** | | **Funding** | | |
| --- | --- | --- | --- | --- | --- | --- | --- | --- | --- | --- | --- | --- | --- |
| **18** | | **19a** | | **19b** | | **20** | | | **21** | | **22** | | |
| **i** | **Score** | **i** | **Score** | **i** | **Score** | **i** | **ii** | **Score** | **i** | **Not included in overall scoring** | **i** | **ii** | **Score** |
| Y | 1 | NA | NA | Y | 1 | N | Y | 0 | N | NA | Y | N | 0 |
| Y | 1 | NA | NA | Y | 1 | N | Y | 0 | N | NA | Y | N | 0 |
| N | 0 | NA | NA | Y | 1 | N | Y | 0 | N | NA | Y | N | 0 |
| Y | 1 | NA | NA | Y | 1 | Y | Y | 1 | N | NA | Y | Y | 1 |
| Y | 1 | NA | NA | Y | 1 | Y | N | 0 | N | NA | N | N | 0 |
| N | 0 | NA | NA | N | 0 | Y | Y | 1 | N | NA | Y | N | 0 |
| N | 0 | NA | NA | Y | 1 | Y | Y | 1 | N | NA | Y | N | 0 |
| N | 0 | NA | NA | Y | 1 | N | N | 0 | N | NA | Y | N | 0 |
| N | 0 | NA | NA | Y | 1 | Y | Y | 1 | N | NA | N | N | 0 |
| N | 0 | NA | NA | Y | 1 | N | N | 0 | N | NA | N | N | 0 |

**Table S2. (cont.)**

**Table S3. Complete risk of bias assessment with PROBAST.**

| **Title** | **Author(s)** | **Participants** | | |
| --- | --- | --- | --- | --- |
|  |  | **Were appropriate data sources used?** | **Were all inclusions and exclusions of participants appropriate?** | **RoB** |
| The Use of Mobile Thermal Imaging and Deep Learning for Prediction of Surgical Site Infection | Fletcher et al. | Y | PY | Low |
| Use of Convolutional Neural Nets and Transfer Learning for Prediction of Surgical Site Infection from Color Images. | Fletcher et al. | PY | PY | Low |
| Application of Machine Learning to Prediction of Surgical Site Infection | Fletcher et al. | PY | PY | Low |
| A Unified Framework for Automatic Detection of Wound Infection with Artificial Intelligence | Wu et al. | NI | NI | Unclear |
| Deepwound: Automated Postoperative Wound Assessment and Surgical Site Surveillance through Convolutional Neural Networks | Shenoy et al. | NI | NI | Unclear |
| Automatic Wound Infection Interpretation for Postoperative Wound Image | Hsu et al. | NI | NI | Unclear |
| Chronic wound assessment and infection detection method. | Hsu et al. | NI | NI | Unclear |
| Implementation of Post-operative Wound Analytics | Zeng et al. | NI | NI | Unclear |
| Photographic LVAD Driveline Wound Infection Recognition Using Deep Learning. | Luneburg et al. | NI | NI | Unclear |
| A unified framework for automatic wound segmentation and analysis with deep convolutional neural networks | Wang et al. | NI | NI | Unclear |

| **Predictors** | | | |
| --- | --- | --- | --- |
| **Were predictors defined and assessed in a similar way for all participants?** | **Were predictor assessments made without knowledge of outcome data?** | **Are all predictors available at the time the model is intended to be used?** | **RoB** |
| PY | NA | NA | Low |
| PN | NA | NA | High |
| PN | NA | NA | High |
| PN | NA | NA | High |
| PN | NA | NA | High |
| PY | NA | NA | Low |
| PY | NA | NA | Low |
| NI | NA | NA | Unclear |
| PY | NA | NA | Low |
| PN | NA | NA | High |

**Table S3. (cont.)**

| **Outcome** | | | | | | |
| --- | --- | --- | --- | --- | --- | --- |
| **Was the outcome determined appropriately?** | **Was a pre-specified or standard outcome definition used?** | **Were predictors excluded from the outcome definition?** | **Was the outcome defined and determined in a similar way for all participants?** | **Was the outcome determined without knowledge of predictor information?** | **Was the time interval between predictor assessment and outcome determination appropriate?** | **RoB** |
| PY | NI | NA | PY | NA | Y | Unclear |
| PY | NI | NA | PY | NA | PY | Unclear |
| PY | NI | NA | PY | NA | PY | Unclear |
| PN | PY | NA | PY | NA | Y | High |
| NI | NI | NA | NI | NA | NI | Unclear |
| PN | NI | NA | PY | NA | PY | High |
| PN | NI | NA | PY | NA | Y | High |
| NI | NI | NA | NI | NA | NI | Unclear |
| PY | NI | NA | PY | NA | NI | Unclear |
| NI | NI | NA | NI | NA | NI | Unclear |

**Table S3. (cont.)**

| **Analysis** | | | | | |
| --- | --- | --- | --- | --- | --- |
| **Were there a reasonable number of participants with the outcome?** | **Were continuous and categorical predictors handled appropriately?** | **Were all enrolled participants included in the analysis?** | **Were participants with missing data handled appropriately?** | **Is all data from a single patient reserved to only a single data partition (e.g. training, testing or tuning)?** | **Was selection of predictors based on univariable analysis avoided?** |
| NI | NA | NI | NA | PY | NA |
| NI | NA | N | NA | PY | NA |
| PN | PY | N | NA | PY | PY |
| NI | NI | PY | NA | NI | NA |
| NI | NA | PY | NA | NI | NA |
| NI | PY | PY | NA | NI | NA |
| NI | PY | PY | NA | NI | NA |
| N | PY | PY | NA | NI | NA |
| NI | NA | N | NA | NI | NA |
| NI | PY | PY | NA | NI | NA |

**Table S3. (cont.)**

| **Analysis** | | | | | **Overall assessment** |
| --- | --- | --- | --- | --- | --- |
| **Were complexities in the data (e.g. censoring, competing risks, sampling of controls) accounted for appropriately?** | **Were relevant model performance measures evaluated appropriately?** | **Were model overfitting and optimism in model performance accounted for?** | **Do predictors and their assigned weights in the final model correspond to the results from multivariable analysis?** | **RoB** | **RoB** |
| Y | NI | PY | NA | Unclear | Unclear |
| Y | NI | PY | NA | High | High |
| PY | NI | PY | NI | High | High |
| PY | NI | PY | NA | Unclear | High |
| PY | NI | N | NA | High | High |
| NI | N | NI | NA | High | High |
| PN | N | NI | NA | High | High |
| NI | NI | PN | NA | High | High |
| PY | N | PY | NA | High | High |
| PN | NI | PY | NA | High | High |

**Table S3. (cont.)**
