# Supplementary material for: Machine Learning Approaches for the Image-Based Identification of Surgical Wound Infections: Scoping Review
Source: J Med Internet Res. 2024 Jan 18;26:e52880. doi: 10.2196/52880 (PMC10835585; doi:10.2196/52880)
Supplement: Multimedia Appendix 6 [file jmir_v26i1e52880_app6.docx]

Fletcher, R. R., Olubeko, O., Sonthalia, H., Kateera, F., Nkurunziza, T., Ashby, J. L., Riviello, R., & Hedt-Gauthier, B. (2019). Application of Machine Learning to Prediction of Surgical Site Infection. *2019 41st Annual International Conference of the IEEE Engineering in Medicine and Biology Society (EMBC)*, 2234–2237. <https://doi.org/10.1109/EMBC.2019.8857942>

Image preprocessing:

- Images were cropped to exclude non-skin parts of the image.
- Each image was scaled to 160×340 pixels.
- Images were converted from RGB to LAB color space.

Feature engineering:

- Gabor wavelet analysis was used to extract textural characteristics.
  - The images were divided into 3×3 blocks, to which Gabor wavelet analysis was applied.
  - Local binary patterns were computed for each pixel.
  - Histograms were generated for each block based on the binary patterns of the pixels in the block.
- Statistical colour analysis was used to extract colour-related features.
  - Each image was divided into 8 blocks.
  - For each block, the mean, variance, and skewness of the pixel values were computed for each colour dimension.

Feature selection:

- L1 regularization was applied to reduce the number of features.
- On average, the model selected 25 features (ranging from 19 to 29 features).

Fletcher, R. R., Schneider, G., Hedt-Gauthier, B., Nkurunziza, T., Alayande, B., Riviello, R., & Kateera, F. (2021). Use of Convolutional Neural Nets and Transfer Learning for Prediction of Surgical Site Infection from Color Images. *Annu Int Conf IEEE Eng Med Biol Soc*, *2021*, 5047–5050.

Image preprocessing:

- For the naïve model, images were not resized or cropped.
- For the transfer learning model, images were resized to 224×224 pixels.
- For both models, data augmentation strategies were undertaken; namely, random flips and random rotations.

Model architectures:

- Naïve model: three convolutional layers with ReLU activation each followed by a pooling layer. These are followed by a fully connected sigmoid activation layer for classification and a dropout layer with a rate of 50%.
- Transfer learning model: ResNet50-initiated model, with an added dense layer with sigmoid activation and an added dropout layer with a rate of 20%.

Fletcher, R. R., Schneider, G., Bikorimana, L., Rukundo, G., Niyigena, A., Miranda, E., Riviello, R., Kateera, F., & Hedt-Gauthier, B. (2021). The Use of Mobile Thermal Imaging and Deep Learning for Prediction of Surgical Site Infection. *Proceedings of the Annual International Conference of the IEEE Engineering in Medicine and Biology Society, EMBS*, 5059–5062. <https://doi.org/10.1109/EMBC46164.2021.9630094>

Image preprocessing:

- The authors used a thermal camera module (SEEK Thermal Compact) that connects to a mobile phone (Samsung Galaxy J8) to obtain a JPG thermal image as well as a 2D temperature array. The 2D temperature arrays were used to create thermal pseudo-images, though it is unclear if these are used, as the authors later indicate that the computer vision script was applied to the “raw thermal image[s]”.
- The authors also used a bespoke mobile app to automatically align, rectify, and crop the images.
- A computer vision script was used to find outline of each wound frame and crop the thermal images. Images were standardized to a size of 160×100 pixels.
- For the transfer learning model, images were scaled to 224×224 pixels.
- For both models, data augmentation strategies were undertaken; namely, random flips and random rotations.

Model architecture:

- Naïve model: three convolutional layers with ReLU activation each followed by a pooling layer. These are followed by a fully connected sigmoid activation layer for classification and a dropout layer with a rate of 50%.
- Transfer learning model: ResNet50-initiated model, with an added dense layer with sigmoid activation and an added dropout layer with a rate of 20%.

Hsu, J.-T., Chen, Y.-W., Ho, T.-W., Tai, H.-C., Wu, J.-M., Sun, H.-Y., Hung, C.-S., Zeng, Y.-C., Kuo, S.-Y., & Lai, F. (2019). Chronic wound assessment and infection detection method. *BMC Medical Informatics and Decision Making*, *19*(1), 99. <https://doi.org/10.1186/s12911-019-0813-0>

Image preprocessing:

- The images are converted to grayscale.
- Otsu’s adaptive thresholding is applied to convert the grayscale images into binary images.

Feature engineering for infection detection:

- To identify points of interest on wound suture sites, the authors detect “cross-shaped features”, which involves applying a cross-shaped kernel to the wound image using morphological operations. Connected-component labelling is used to connect adjacent pixels which are grouped into clusters, which represent wound feature points.
- To identify regions of interest (ROIs), which consist of various grouped feature points, the authors use hierarchical clustering. To determine the optimal clustering number, the authors calculate the silhouette score for each hierarchy and examine how the score changes with the number of clusters using data from multiple wounds. Past a certain number of clusters, the authors find that the score begins to converge. To identify this threshold, the authors use the inverse of this score, noting that it is an empirically unimodal function, and use a unimodal thresholding algorithm. The authors identify the minimum upright bounding rectangle for each cluster to detect the ROIs.
- Feature vectors are calculated for four wound symptoms: namely, swelling, granulation, infection, and necrosis. The specific steps are provided only for swelling, which essentially consist of defining specific hue ranges for (non-swollen) skin and different forms of swelling, isolating the hue information from the image, and calculating mean values for pixel histograms.

Hsu, J.-T., Ho, T.-W., Shih, H.-F., Chang, C.-C., Lai, F., Wu, J.-M., Hsu, J.-T., Ho, T.-W., Shih, H.-F., Chang, C.-C., Lai, F., & Wu, J.-M. (2017). Automatic wound infection interpretation for postoperative wound image. *SPIE*, *10225*, 1022526. <https://doi.org/10.1117/12.2266110>

- Same as above (Hsu et al., 2019).

Lüneburg, N., Reiss, N., Feldmann, C., van der Meulen, P., van de Steeg, M., Schmidt, T., Wendl, R., & Jansen, S. (2019). Photographic LVAD Driveline Wound Infection Recognition Using Deep Learning. *Studies in Health Technology and Informatics*, *260*, 192–199.

Image preprocessing:

- An algorithm for detecting out-of-focus images was developed using the Laplacian operator, with an empirically/manually derived threshold from a set of 692 images. Out-of-focus images were filtered out.
- Data augmentation strategies (affine transformations) were used to enhance the training of the U-Net models.

Segmentation:

- The authors compared two approaches to driveline tube segmentation, an unsupervised approach and a supervised approach:
  - The Felzenszwalb unsupervised segmentation algorithm, which groups adjacent pixels based on global and local contrast, serving to merge similar regions.
  - A supervised deep learning approach (using U-Net) that leverages manually annotated images for the ground-truth segmentation map.

ROI:

- To identify a region of interest, the authors trained a U-Net to detect the area around the driveline exit site. These regions are converted into rectangular sections and used as input for classification.

Infection detection:

- The authors test various CNN architectures, finding that the best-performing architecture is the VGG-16 architecture pretrained on ImageNet, with additional fine-tuning using their own images.

Shenoy, V., Foster, E., Aalami, L., Majeed, B., & Aalami, O. (2018). Deepwound: Automated Postoperative Wound Assessment and Surgical Site Surveillance through Convolutional Neural Networks. *ArXiv*. <https://doi.org/10.48550/arxiv.1807.04355>

Image preprocessing:

- The authors apply contrast-limited adaptive histogram equalization (CLAHE) to adjust for differences in lighting.
- Data augmentation strategies, including random rotation, flips, zooms, shears, and shifts, are applied prior to training.

Model architecture:

- The authors create three different CNNs which use a slightly modified VGG-16 architecture pretrained with ImageNet. The difference between these three ‘WoundNet’ architectures is that one of them is frozen at layer 6, one is frozen at layer 10, and one is frozen at layer 14. The modifications consist of removing the final output layer as well as two fully connected (FC) layers containing 4096 neurons from the original VGG-16, replacing them with a sigmoid activation output layer and two smaller FC layers.
- The authors train the CNNs using backpropagation with a batch size of 64 and the binary cross-entropy loss function. Two different optimizers are used: the Adam optimizer is initially used for 30 epochs (learning rate of 1e-3), followed by stochastic gradient descent for 50 epochs (learning rate of 1e-4).
- The authors create an ensemble of the three CNNs called Deepwound.

Wang, C., Yan, X., Smith, M., Kochhar, K., Rubin, M., Warren, S. M., Wrobel, J., & Lee, H. (2015). A unified framework for automatic wound segmentation and analysis with deep convolutional neural networks. *Proceedings of the Annual International Conference of the IEEE Engineering in Medicine and Biology Society, EMBS*, *November 2015*, 2415–2418. <https://doi.org/10.1109/EMBC.2015.7318881>

Wound segmentation:

- The authors use a convolutional encoder-decoder network to generate a binary mask for the region of the image that corresponds to a wound. The encoder network generates feature maps which are upsampled by the decoder network to generate the segmentation masks.
- The surface area of the segmented wound is estimated using the pixel area and ruler ticks in the image. Hough transform is applied to parametrize the ruler ticks.

Wound infection:

- The authors use the features learned in the segmentation step to train an SVM classifier.

Healing prediction:

- The authors seek to create a model that can predict future wound surface areas using the wound images and their corresponding surface areas. To this end, the authors use Gaussian process regression to model the relationship between the encoder-learned features and the surface areas.

Wu, J.-M., Tsai, C.-J., Ho, T.-W., Lai, F., Tai, H.-C., & Lin, M.-T. (2020). A Unified Framework for Automatic Detection of Wound Infection with Artificial Intelligence. *Applied Sciences*, *10*(15), 5353. <https://doi.org/10.3390/app10155353>

Image preprocessing:

- A log transform followed by a gamma transform were applied to the images.
- Eight colours on the colour cards were used for colour calibration.

Model architectures:

- A ResNet50-initiated CNN was used to identify the presence of colour cards in the wound images. This CNN was modified by replacing the global average pooling layer and fully connected layer with an additional convolutional layer and a bilinear upsampling 2D layer (to resize the feature maps to a defined dimension). This model was fine-tuned using the PASCAL VOC 2012 database.
- The Speed-Up Robust Feature method and the Fast Library for Approximate Nearest Neighbours were used to extract and match specific points on the colour card and the photographed colour card.
- For infection detection, the authors used Xception, a CNN. Additional classification methods were also tested (SVM, random forest, and gradient boosting), but details are not provided.

Zeng, Y. C., Liao, K. H., Wang, C. H., Lin, Y., & Chang, W. T. (2017). Implementation of post-operative wound analytics. *2017 IEEE International Conference on Consumer Electronics - Taiwan, ICCE-TW 2017*, 93–94. <https://doi.org/10.1109/ICCE-CHINA.2017.7991011>

Wound detection:

- To define the wound area, Difference of Gaussians (DoG) is applied to the wound images. Gaussian smoothing is then further applied to the modified images. The authors classify parts of the image as wound areas or non-wound areas using a fixed threshold (calculated using the mean and standard deviation of the difference between the DoG-filtered image and the Gaussian-smoothed image).
- The images are colour-normalized using the average RBG values of the non-wound area (as ascertained in the prior step) and empirically defined RBG values representative of skin.
- The authors identify skin and non-skin areas of the image (details are not provided; they cite a 2003 paper that describes three methods for detection of skin colour pixels—the authors likely employed the method that uses the RGB colour space, which specifies a set of simple thresholds for a pixel to be considered as having skin colour).
- To identify which skin areas are wound areas based on colour, the authors calculate the ratio of skin-colour pixels to non-skin-colour pixels. If the ratio exceeds a threshold (r > 2), the non-skin-colour pixels within the skin area is considered a wound area.
- To determine the final wound area, the authors consider both edge/texture-based wound areas and colour-based wound areas.

Infection detection:

- For feature extraction, the colour-normalized image is divided into overlapping blocks, and both histogram-based features and gray-level co-occurrence matrix (GLCM) features are computed for each block.
- An SVM classifier is trained on these features. At test time, the algorithm focuses on blocks that overlap with the wound area.
